# High-throughput phase elucidation of polycrystalline materials using serial rotation electron diffraction

**DOI:** 10.1038/s41557-022-01131-8

**Published:** 2023-01-30

**Authors:** Yi Luo, Bin Wang, Stef Smeets, Junliang Sun, Weimin Yang, Xiaodong Zou

**Affiliations:** 1 Department of Materials and Environmental Chemistry, Stockholm University, Stockholm, Sweden; 2 State Key Laboratory of Green Chemical Engineering and Industrial Catalysis, Sinopec Shanghai Research Institute of Petrochemical Technology, Shanghai, China; 3 Netherlands eScience Center, Amsterdam, Netherlands; 4 College of Chemistry and Molecular Engineering, Beijing National Laboratory for Molecular Sciences, Peking University, Beijing, China

**Keywords:** Analytical chemistry, Synthetic chemistry methodology, Materials chemistry, Computational chemistry

## Abstract

Rapid phase elucidation of polycrystalline materials is essential for developing new materials of chemical, pharmaceutical and industrial interest. Yet, the size and quantity of many crystalline phases are too small for routine X-ray diffraction analysis. This has become a workflow bottleneck in materials development, especially in high-throughput synthesis screening. Here we demonstrate the application of serial rotation electron diffraction (SerialRED) for high-throughput phase identification of complex polycrystalline zeolite products. The products were prepared from a combination of multiple framework T atoms ([Si,Ge,Al] or [Si,Ge,B]) and a simple organic structure-directing agent. We show that using SerialRED, five zeolite phases can be identified from a highly complex mixture. This includes phases with ultra-low contents undetectable using X-ray diffraction and phases with identical crystal morphology and similar unit cell parameters. By automatically and rapidly examining hundreds of crystals, SerialRED enables high-throughput phase analysis and allows the exploration of complex synthesis systems. It provides new opportunities for rapid development of polycrystalline materials.

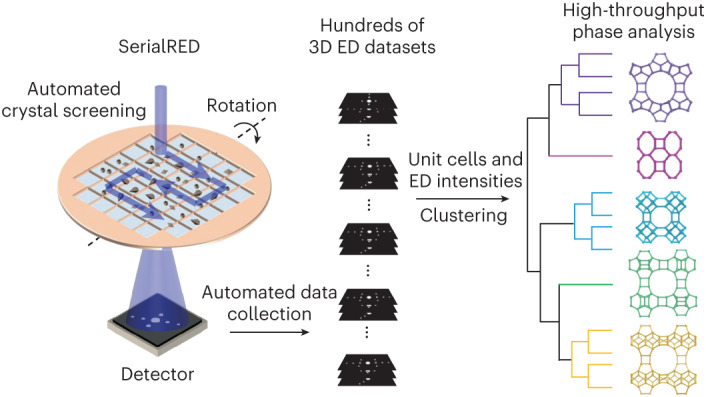

## Abstract (web summary)

X-ray diffraction is crucial for the phase elucidation of polycrystalline materials but remains challenging for complex multiphase systems. Now serial rotation electron diffraction has been shown to enable rapid, reliable and semiquantitative phase analysis of such systems, facilitating high-throughput screening of complex synthesis systems and providing new opportunities for materials development.

## Main

Many crystalline materials are widely used in various applications, because of their optical, electrical, thermal, mechanical and/or magnetic properties. The development of materials with specific properties continues to attract significant interest. Innovative synthetic routes are being developed but products are often formed as polycrystalline materials with crystals too small or too complex to be studied using routine X-ray diffraction methods^1–3^. There is a great demand for new techniques that can unravel the phase compositions and atomic structures reliably at an early stage in the design of novel materials. These are essential for rationalizing the synthesis, evaluating properties and steering the design for new promising applications of the materials^1,4–8^. In addition, recent trends in applying high-throughput synthesis techniques for materials screening can result in numerous polycrystalline products in a short time.^9–16^ Typical workflows of high-throughput syntheses require rapid and reliable phase elucidation, but for polycrystalline materials this has remained a major challenge^17^.

Since its first discovery over a century ago, X-ray diffraction has been well-established for phase analysis and structure determination. Single-crystal X-ray diffraction (SCXRD) is the standard technique to obtain accurate crystal structures for large single crystals (larger than about 5 × 5 × 5 μm^3^ for a laboratory instrument). Applying this large-single-crystal-based technique to phase analysis of polycrystalline materials is challenging because of the small crystal sizes and the mixture of different phases. Alternatively, powder X-ray diffraction (PXRD) is routinely used, but has several major limitations. Reflections with equal or similar *d* spacings overlap in the PXRD pattern, making the phase and structure identification of polycrystalline materials by PXRD difficult and time consuming, and sometimes impossible^17–19^. Challenges arise when a polycrystalline product contains (1) multiple phases, (2) phases with ultra-low contents (<1%), (3) phases with similar unit cell parameters and/or (4) structures with large unit cells or low symmetries which result in more peaks and peak overlaps in PXRD patterns^20^. Some interesting crystalline materials may therefore be easily overlooked or discarded.

**Fig. 1:**
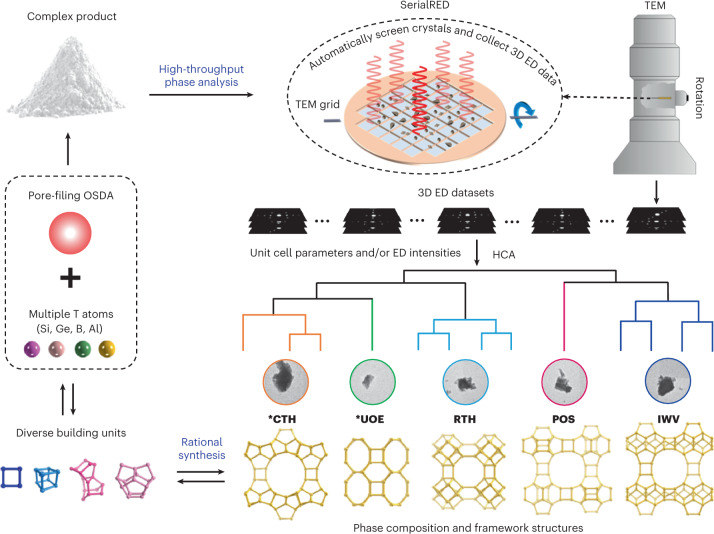
Exploration of complex polycrystalline zeolite products via high-throughput phase identification using SerialRED. A complex synthesis system consisting of multiple framework T atoms ([Si,Ge,Al] or [Si,Ge,B]) and a simple OSDA was designed to synthesize zeolites. The different framework T atoms were expected to trigger the formation of a diverse variety of building units and result in various framework structures, and the OSDA mainly plays a pore-filling role. In each case, the product was obtained as a complex polycrystalline powder, whose phase composition was then studied by SerialRED. SerialRED automatically screens hundreds of crystals on a TEM grid and collects a 3D ED dataset on each crystal. The unit cell parameters obtained from the 3D ED datasets of a large number of crystals were clustered and assigned to each zeolite phase (*CTH, *UOE, and so on) via HCA, where the Euclidean distances between the unit cell parameters were used as a metric. The diffraction intensities of the 3D ED datasets of each zeolite phase were then further clustered based on the correlations of their diffraction intensities. The datasets within the same cluster were merged and used for structure determination. The detailed phase and structural information allowed us to understand the correlations between the framework T atoms, building units and framework structures, which enables the rational development of zeolite materials.

Electrons, on the other hand, interact ∼10^4^ times more strongly with matter than X-rays do^21,22^, enabling useful single-crystal electron diffraction data from crystals with sizes down to 50 nm (refs. ^23,24^). Three-dimensional electron diffraction (3D ED) is analogous to SCXRD, and has the advantage of enabling the study of crystals at nano- and micrometre scales. The rapid developments of 3D ED over the past decade have allowed researchers to discover the benefits of 3D ED for phase identification and structure determination of polycrystalline materials that are too challenging to be studied by SCXRD/PXRD^25–36^. With 3D ED, electron diffraction data are collected from a single crystal of an arbitrary orientation while the crystal is rotated around an axis, either in a continuous rotation or by stepwise rotation combined with precession or beam tilt^37–41^. However, searching crystals for 3D ED data collection is still mostly a manual and time-consuming endeavour. The selection of crystals for data collection can be subject to human bias and as a result some of the phases may be missed. Although over thousands of crystals are often available on an electron microscopy grid, only a handful of crystals can be measured during a typical electron microscopy session (for example, 4 h). Recently, Smeets et al. developed a serial electron diffraction (SerialED) method that applies a snapshot data collection strategy to 3D ED for structure determination and phase analysis of polycrystalline materials^42,43^. Bücker et al. then demonstrated the advantages of SerialED in protein nanocrystallography^44^. The fully automated protocol of SerialED eliminates human intervention in crystal searching and data collection. Based on the SerialED protocol, Wang et al. developed the high-throughput SerialRED method, which automatically screens crystals and collects 3D ED data from hundreds of crystals in a product^45^. It combines phase analysis and structure determination in a single technique. Combined with hierarchical cluster analysis (HCA), SerialRED enables objective, high-throughput phase analysis and structure determination of products with multiple phases and nanometre- and submicrometre-sized crystals (Fig. 1). This makes SerialRED an ideal technique for high-throughput synthesis screening, facilitating development of novel polycrystalline materials.

Zeolites are a class of typically metastable polycrystalline microporous materials that are widely applied in industry^46^. They are often naturally formed as submicrometre-sized crystals with complex structures and are frequently synthesized as multiphases. This makes phase identification and structure analysis challenging^17^. In this work, we demonstrate the use of SerialRED for high-throughput phase identification of zeolite synthesis products through a real example in zeolite material exploration (Fig. 1). We combine multiple tetrahedrally coordinated framework T atoms (T = Si, Ge, Al or B) and a simple organic structure-directing agent (OSDA) to synthesize a variety of zeolite materials. In the synthesis, the Si/Ge molar ratios were varied from 5 to 15 accompanied with the (Si + Ge)/T^III^ (T^III^ = Al or B) molar ratios ranging from 5 to 100 to screen zeolite materials. By changing the ratios of different types of T atoms in the synthesis batches, a large number of products are obtained, many as phase mixtures. The phase compositions of the resulting products were initially characterized by PXRD. Products that could not be identified with PXRD or conventional 3D ED were then investigated by SerialRED through high-throughput phase identification. Here we choose the most complex sample (product A: Si/Ge = 10, (Si + Ge)/Al = 15) obtained during the zeolite exploration to demonstrate the capability of SerialRED in phase analysis of complex polycrystalline products. By combining SerialRED with HCA, we identified five zeolite phases in the sample. Among the five zeolite phases, two have ultra-low contents that are not detected by PXRD, and two have identical crystal morphology and similar unit cell parameters. We used another sample (product B: Si/Ge = 5, (Si + Ge)/Al = 12.5) to show it is possible to identify phases with similar crystal morphology and unit cell parameters, and to demonstrate the potential of quantitative phase analysis using SerialRED. These show the advantage of SerialRED in rapidly accessing reliable phase information in a high-throughput way where conventional methods fall short. With the phase information identified by SerialRED, the roles of different framework T atoms were disclosed, which offers more opportunities for the rational synthesis of zeolite materials. We also show the proposed approach is promising for large-scale synthesis of large and extra-large pore zeolite materials for industrial applications.

## Results and discussion

### Synthesis and phase diagram

In Table 1, we show the synthesis and subsequent identification of zeolite phases in multiphase materials synthesized from multiple framework T atoms ([Si,Ge,Al] or [Si,Ge,B]) and a pore-filling OSDA (4-dimethylaminopyridine, DMAP). The details of the synthesis experiments are given in Methods. The OSDA has previously been used for the synthesis of zeolites with **SFO**, **POS** and *UOE type frameworks (the three uppercase bold letters are the framework type codes of the zeolites; an asterisk indicates the framework is disordered)^47–50^. Studies have shown that the combination of framework T atoms directs the formation of specific structure-building units^51^. Among them, silicon and germanium ([Si,Ge]) are mostly combined together to synthesize large or extra-large pore zeolites that are normally unstable and lack active sites^52^. The addition of aluminium or boron into the [Si,Ge] system usually triggers an increase in the diversity of structure-building units (Fig. 1), introduces active sites and results in thermally more stable large or extra-large pore zeolites^51^. By tuning the Si/Ge molar ratios from 5 to 15 and the (Si + Ge)/T^III^ (T^III^ = Al or B) molar ratios from 5 to 100, most synthesis batches give rise to crystalline products, including five pure phases and a series of mixtures of biphases or multiphases (Table 1 and Supplementary Figs. 1 and 2). In the [Si,Ge] system ((Si + Ge)/T^III^ = ∞), two framework types, **TON** (1D, 10-ring) and **POS** (3D, 12 × 11 × 11-ring) were obtained (Table 1 and Supplementary Figs. 1 and 2)^53^. With the gradual introduction of boron into the [Si,Ge] system, framework types **NON** (0D) and **SFE** (1D, 12-ring) were obtained (Table 1 and Supplementary Figs. 1 and 2), while the introduction of aluminium into the [Si,Ge] system triggers the formation of **NON** and **RTH** (2D, 8 × 8-ring) and a series of multiphase products^54–57^.

**Table 1:** Synthesis parameters and corresponding phases for the studied samples^a^

		OSDA/Si = 0.6, HF/Si = 0.6, H_2_O/Si = 10		
		Si/Ge = 15	Si/Ge = 10	Si/Ge = 5
(Si + Ge) (no T^III^ atoms)		**TON**	**TON**, **POS**	**POS**
(Si + Ge)/Al	100	**NON**	*Amorphous*, *dense*, *UOE	*Amorphous*, *dense*, *UOE
	20	Amorphous	*Amorphous*, ***RTH***, ***IWV***, *CTH	***RTH***, *UOE, ***POS***, ***IWV***, *CTH
	15	*Amorphous*, *dense*, *UOE, ***IWV***	*Dense*, ***RTH***, *UOE, ***POS***, ***IWV***, *CTH	***RTH***, *UOE, ***POS***, ***IWV***, *CTH
	10	**RTH**	***RTH***, *UOE, ***POS***, ***IWV***, *CTH	***RTH***, ***IWV***, *CTH
	5	Amorphous	Amorphous	**RTH**
(Si + Ge)/B	100	**NON**	Amorphous	Amorphous, **POS**
	20	Amorphous, **SFE**	**SFE**	**SFE**, **TON**
	15	Amorphous, **SFE**	**SFE**	**SFE**, **TON**
	10	**SFE**	**SFE**	**SFE**
	5	**SFE**	**SFE**	SFE

^a^The samples were synthesized hydrothermally at 170 °C for 10 days using DMAP as the OSDA. T^III^ = Al or B; dense = dense phase (SiO_2_/GeO_2_). The products in italic are complex phase mixtures that PXRD could not identify.

**Fig. 2:**
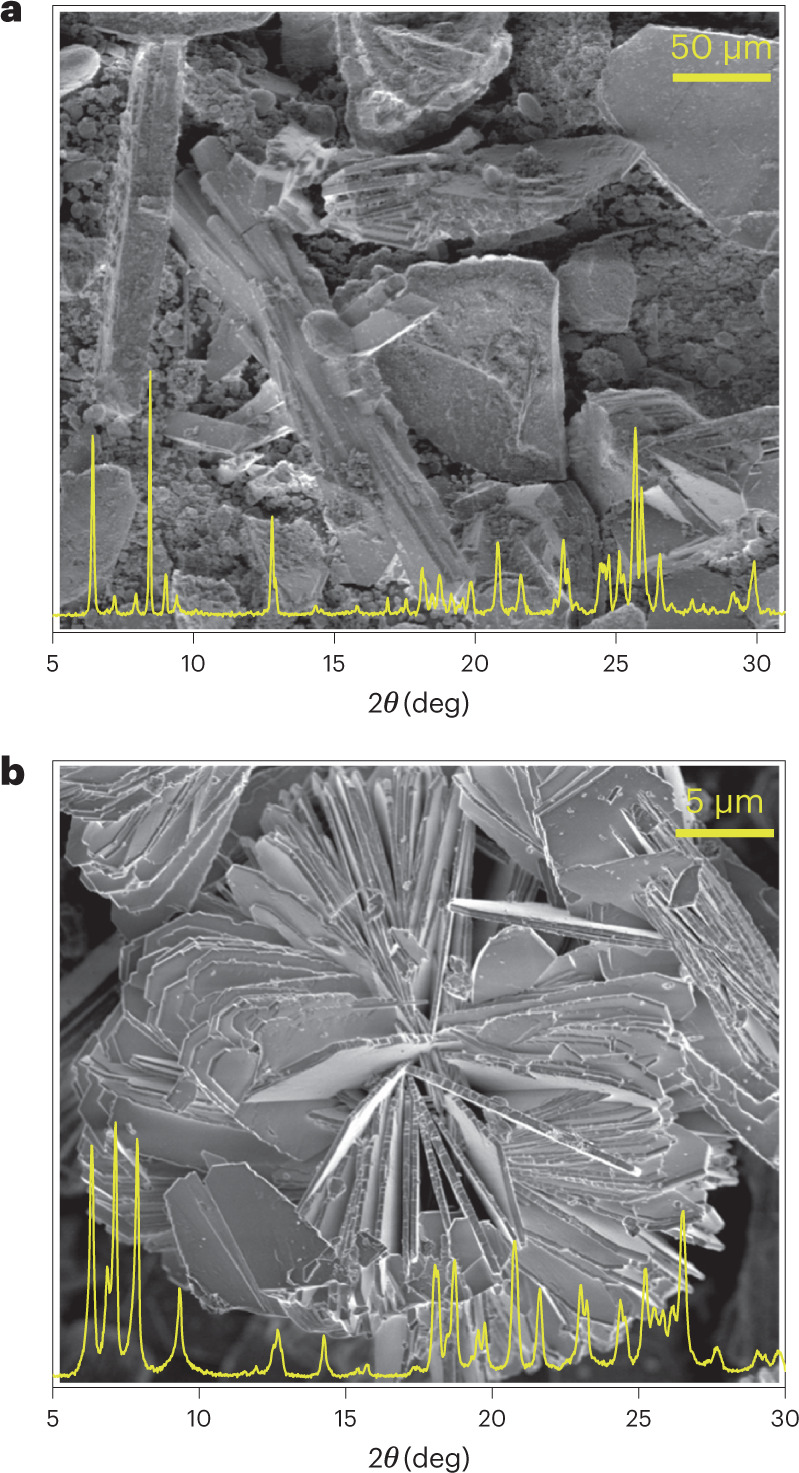
PXRD patterns and SEM images. **a**, Product A. **b**, Product B. Crystals with needle- and plate-like morphologies are observed in product A (Si/Ge = 10, (Si + Ge)/Al = 15). Uniform plate-like crystals are present in product B (Si/Ge = 5, (Si + Ge)/Al = 12.5). More details (original PXRD patterns and additional SEM images) of product A and product B are given in Supplementary Figs. 3 and 5, respectively. Source data

The phases of these pure products, and of most biphase products with large differences in unit cells or morphologies, were identified by PXRD in combination with scanning electron microscopy (SEM) (Supplementary Figs. 1 and 2). However, for some of the biphase products (with similar unit cells or morphologies) and most of the multiphase products, only part of the phase information could be identified by PXRD (as highlighted in italics in Table 1). Figure 2a presents the PXRD pattern and SEM image of a typical complex product with mixed phases (denoted as product A). In product A, only a significant **RTH** (needle-like) component was identified by PXRD (Supplementary Fig. 4a). SEM shows the presence of crystals with both needle- and plate-like morphologies (Fig. 2a and Supplementary Fig. 3). Therefore, product A was initially thought to contain two phases, the needle-like **RTH** phase and an unknown phase with the plate-like morphology. A two-step heating programme (at 110 °C for 1 day and then 170 °C for 5 days) was applied to promote the crystallization of the plate-like crystals and avoid the formation of **RTH** (Synthesis of zeolite materials in Methods). SEM showed that the resulting material (denoted product B) contains uniform plate-like crystals (Fig. 2b and Supplementary Fig. 5), and therefore product B was regarded initially as a pure phase. However, despite its high crystallinity (Supplementary Fig. 7), we were unable to index the PXRD pattern using the SVD-index method implemented in TOPAS v.6^58^.

Therefore, we turned to conventional 3D ED, more specifically continuous-rotation electron diffraction (cRED), a technique that enables us to collect high-quality 3D ED data for an isolated submicrometre-sized single crystal^31,41^. This showed the presence of zeolite **IWV** (2D, 12 × 12-ring, plate-like crystal) in product B (Supplementary Fig. 6)^4,59^. Subsequently, **IWV** was also identified in product A by comparing the PXRD patterns of product A and product B (Fig. 2) and the Pawley profile fit of the PXRD pattern with both **RTH** and **IWV** (Supplementary Fig. 4b). However, some peaks in the PXRD pattern of product B could not be indexed with only the unit cell of **IWV** (Supplementary Fig. 7). When we tried to fit the PXRD pattern of product A with two phases, **RTH** and **IWV**, there were still a few unindexed peaks (Supplementary Fig. 4b). Therefore, some of the phases in these two products may still be missed by PXRD analysis and even by conventional 3D ED study.

### High-throughput phase identification using SerialRED

To systematically tackle the problem and identify all the phases in product A and product B, we used the SerialRED method^45^. SerialRED enables us to collect 3D ED data from hundreds of individual crystals automatically, with the aim of performing high-throughput phase analysis and structure determination. The SerialRED experiments were performed using the protocol implemented in the software Instamatic and on a trace amount of sample^41,45^. For product A, the SerialRED routine ran for 6 h, resulting in 321 3D ED datasets (Supplementary Fig. 8). In our set-up, we have integrated the program DIALS for on-the-fly unit cell determination for each dataset^60^. As a result, 146 datasets were indexed with the corresponding unit cell parameters. Out of these, 77 datasets with a rotation range larger than 20° were used for phase analysis. The high-throughput phase analysis was performed by loading the identified unit cells into the HCA algorithm implemented in the package edtools (Supplementary Fig. 9)^45,61,62^. The Euclidean distance (equation (1) in Methods) between the unit cell parameters was used as the metric for the HCA. The datasets within each cluster were used for another round of intensity-based HCA to select the datasets with the highest correlation (to remove poor-quality datasets and outliers) and merge those for structure analysis (Supplementary Fig. 9; more details are given in Methods)^61^.

**Fig. 3:**
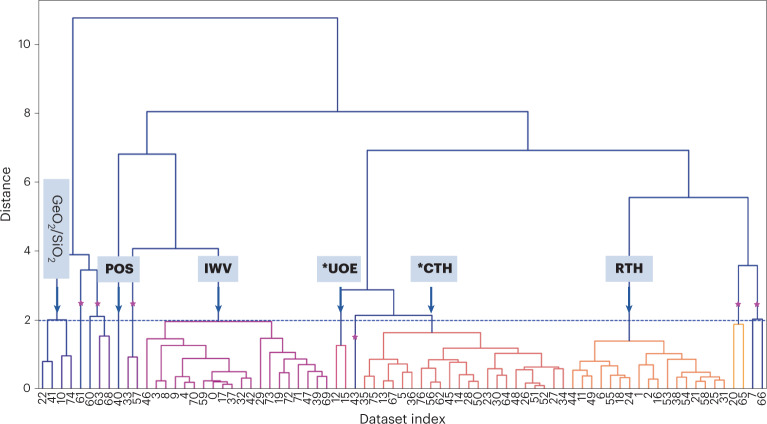
Dendrogram showing the results of the HCA of product A. The *y* axis is the Euclidean distance between the unit cell parameters and is described in Methods (equation (1)). The horizontal axis is the index of the 3D ED datasets used for HCA. HCA showed five zeolite phases (highlighted by arrows), **RTH**, **IWV**, *CTH, *UOE and **POS**, by setting the Euclidean distance cut threshold at 2.0. The branches under each phase/cluster are of the same colour. As indicated by the number of branches (one branch represents one crystal) under each phase, **RTH**, **IWV** and *CTH are the major phases, and **POS** and *UOE are the minor phases. The unclassified datasets (marked by a purple star) could not be identified. These could be data from crystal agglomerates or of otherwise poor data quality, which both result in inaccurate unit cell parameters with large deviations (Supplementary Fig. 11). The unit cell parameters of all datasets for the HCA are given in Supplementary Table 1.

Figure 3 and Supplementary Table 1 present the HCA results of product A. The HCA showed the presence of five zeolite framework types, including three more phases *UOE (2D, 10 × 8-ring, needle-like), **POS** (needle-like) and *CTH (2D, 14 × 12-ring, plate-like)^63^, in addition to **RTH** (needle-like) and **IWV** (plate-like) (Supplementary Tables 1 and 2) which were already identified by combining PXRD and cRED. Among the five zeolites, **RTH**, **IWV** and *CTH were the major phases, and *UOE and **POS** were the minor phases as indicated by the number of crystals clustered in each phase. The selected 3D ED datasets for each phase were merged, and the framework structures could be determined directly by SHELXT (Fig. 3, Supplementary Fig. 10 and Supplementary Table 3)^64^. These findings are corroborated by the Pawley fit of the PXRD pattern of different phases using the routine implemented in the program TOPAS v.6. The PXRD pattern of product A can be well fitted with phases **RTH**, **IWV** and *CTH, which confirms that they are the major phases in product A (Supplementary Fig. 4c). For *UOE and **POS**, which have almost identical morphologies to **RTH** (Fig. 2 and Supplementary Fig. 2), no obvious reflections from them are observed, and no significant improvement of the Pawley fit is obtained after including these phases (Supplementary Fig. 4c,d). This clearly highlights the great advantage of SerialRED in picking up minor phases that could not be detected by PXRD and/or distinguished by SEM.

In product B, the minor phase *CTH, which has a similar crystal size and morphology as **IWV**, was also identified by SerialRED (Supplementary Figs. 12–14 and Supplementary Table 4). Notably, two of the unit cell dimensions of **IWV** and *CTH are very similar (Supplementary Table 2). *CTH was not detected using conventional 3D ED, which can be attributed to a combination of the small number of crystals sampled and crystal selection bias. The larger number of crystals sampled by SerialRED provides improved statistics and more objective crystal selections on the phase information. HCA of the SerialRED datasets from product B shows that 27 crystals of **IWV** and 9 crystals of *CTH (75% **IWV**, 25% *CTH; Supplementary Fig. 13 and Supplementary Table 4) were detected. Rietveld refinement of product B against the synchrotron powder X-ray diffraction (SPXRD) data using TOPAS v.6 (Supplementary Fig. 15 and Supplementary Table 6) gave a phase composition of 67% **IWV**, 33% *CTH. The metrics differ in that SPXRD Rietveld refinement determines weight percent and HCA counts the number of crystals. This indicates that SerialRED is capable of quantitative phase analysis for crystalline materials as suggested previously, as long as the number of crystals collected is large enough^45^.

### SerialRED for the development of zeolite materials

**Fig. 4:**
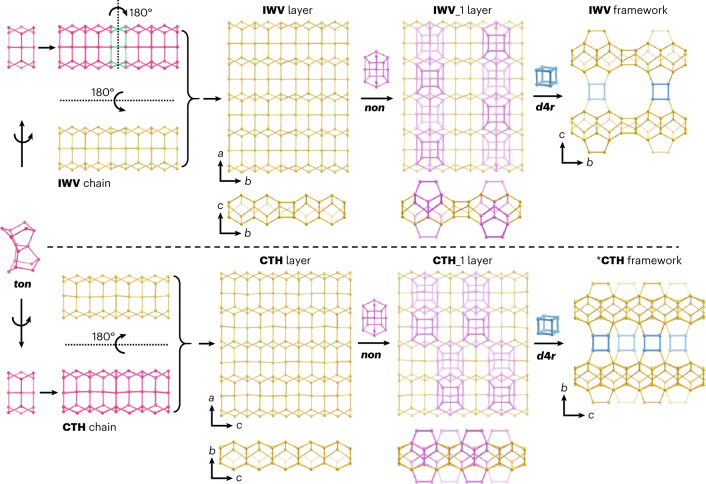
Structural relationship between IWV and *CTH. The frameworks of **IWV** and *CTH are highly related and both can be constructed by *ton*, *non* and *d*4*r* building units in a very similar manner. The very similar **IWV** and **CTH** layers, which are also closely related to those of **TON** and **NON** (Supplementary Fig. 16), are built from *ton* units. The incorporation of *non* units to the **IWV** and **CTH** layers results in the **IWV**_1 layer and **CTH**_1 layer, respectively. The neighbouring **IWV**_1 layers and **CTH**_1 layers are then connected by sharing *d*4*r* units to form the **IWV** and *CTH frameworks, respectively. Only one type of the ordered *CTH framework is presented here. Oxygen atoms have been omitted from the structures for clarify.

With the complete information of the phases present in the sample as identified by SerialRED, the roles of different framework T atoms Si, Ge, Al and B in the synthesis system became clear. We noticed that the framework structures of **NON**, **TON**, **SFE**, **IWV** and *CTH, synthesized from the proposed system using the same OSDA (DMAP), are all highly related and contain similar building chains (**TON**, **NON**, **IWV** and **CTH** chains) and layers (**TON**, **NON**, **IWV** and **CTH** layers). Those chains and layers consist of similar building units (*ton* or *5*^*2*^*6*^*2*^; Fig. 4 and Supplementary Fig. 16). Based on the common structural features of those zeolites and their synthesis conditions, the formation of the corresponding chains, layers or building units can be attributed to a silicon-based system with a small amount of germanium (Fig. 4 and Supplementary Fig. 16). Meanwhile, the differences in the framework structures and synthesis conditions show that the formation of *non* and *d*4*r* units were triggered by a small amount of boron or aluminium and a considerable amount of germanium, respectively. Therefore, the syntheses of **IWV** and *CTH (both of which contain *ton*, *non* and *d*4*r* units) were promoted by introducing considerable aluminium and germanium into the synthesis system of **TON** (Fig. 4). The close structural relationship between **IWV** and *CTH also explains the difficulty in synthesizing pure phases of **IWV** and *CTH. Introduction of significant boron into the synthesis system of **TON** resulted in the zeolite **SFE**, whose framework structure is related to that of **TON** through *σ* expansion (Supplementary Fig. 17). This clearly shows the role of boron in promoting the formation of small *s*4*r* units^51^. The syntheses of zeolites **SFE**, **IWV** and *CTH typically require bulky and expensive organic molecules as the OSDAs (Supplementary Table 7). In our designed synthesis system, **SFE**, **IWV** and *CTH were successfully synthesized using a simple, commercially available DMAP as the OSDA, which is therefore economically more viable for large-scale production. It is also worth mentioning that Yang et al. obtained *UOE and *CTH as a phase mixture in 2015^65^, before the first reports of *UOE and *CTH in 2016 and 2018, respectively^50,63^. At that time, the product was too complex to be analysed and was thus discarded. If SerialRED had been available then, they could have used the technique to identify those novel phases and solve the structures. Therefore, we believe SerialRED can make important contributions to identify interesting materials at an early stage of the synthesis. The proposed approach is promising for large-scale production of targeted zeolite materials for industrial applications. SerialRED can be a powerful and automated screening method for the exploration of such complex synthesis systems.

### Catalysis

The phase information is important for understanding the catalytic properties of zeolites. We conducted a catalysis test of product B, a mixture of two zeolites with the **IWV** (67%) and *CTH (33%) frameworks, on the isomerization of bulky isopropylnaphthalene (IPN) molecules. The reaction is used for preparation of 2,6-diisopropylnaphthalene, an important monomer for production of advanced polyester fibres, films and plastics (Supplementary Figs. 18–22 and Supplementary Tables 8 and 9)^66,67^. Its performance was compared with those of **MOR** (2D, 12 × 8-ring) and **SFE** (1D, 12-ring) zeolites, which are excellent catalysts for the isomerization of IPN^67–70^. The results show that product B with moderate acid properties has a much higher catalytic efficiency than **MOR** and **SFE** (Supplementary Figs. 21 and 22 and Supplementary Table 9). This indicates that product B has more accessible active sites for IPN than **MOR** and **SFE** have. This is because product B contains two 2D large-pore (**IWV**, 12 × 12-ring) and extra-large-pore (*CTH, 14 × 12-ring) zeolites, which facilitates diffusion of bulkier molecules. These findings offer detailed insights in developing novel applications of this catalyst in industrial processes that involve bulky molecules such as oil refining and fine chemical synthesis.

## Conclusion

In this work, using a real example in the exploration of zeolite materials, we have demonstrated the benefit of SerialRED for characterizing and developing polycrystalline materials. Through its ability to automatically screen a large number of single crystals and collect 3D ED data, SerialRED offers new opportunities for rapidly accessing reliable phase information for complex polycrystalline products via high-throughput screening. Five zeolites, **RTH**, *UOE, **POS**, **IWV** and *CTH, some of which had ultra-low content, similar morphology and unit cell parameters that could not be detected/identified by PXRD or even by conventional 3D ED, were identified by SerialRED in a highly complex zeolite mixture. The ability of SerialRED to identify interesting phases at an early stage in the synthesis development provides unique opportunities to understand the role of different framework T atoms and to accelerate the development of zeolite materials. We also demonstrate the potential of quantitative phase analysis using SerialRED data which showed general agreement with the results obtained from PXRD data. In addition, the SerialRED experiments are performed on a trace amount of sample, which is desirable for nanomole-scale high-throughput synthesis screening. These unique advantages of SerialRED substantially expand the scope of synthetic chemistry for the discovery of interesting polycrystalline materials. In addition to studying zeolites, SerialRED can also facilitate the exploration of a wide range of materials, from minerals, metal/metal oxides, ceramics and semiconductors to organic and pharmaceutical compounds, and studies of their polymorphism.

## Methods

### Design of synthesis experiments

Extensive studies have demonstrated that OSDAs play a core role in the crystallization of zeolites. In recent years, the search for novel zeolite materials has been driven by the design and synthesis of novel OSDAs, endowing the molecular structures of OSDAs with large sizes, unique conformations and compositions, and a multiplicity of functions^71^. Large, bulky OSDAs usually show strong structure-directing ability and can dominate the crystallization process to form specific framework structures^72^. Many novel zeolites have therefore been synthesized using large, bulky OSDAs. However, the preparation of such OSDAs is always challenging, complex and costly, which has limited their use for large-scale preparation of novel zeolites.

Here, instead of using large, bulky OSDAs, we investigate the utilization of multiple framework T atoms (T = Si, Ge, Al, B) to synthesize zeolite materials. Many studies have shown that different framework T atoms demonstrate preferences in directing the formation of specific structure-building units under certain conditions due to the difference in T–O bond lengths and T–O–T bond angles (Supplementary Table 10).^51,73,74^ The substitution of silicon by boron, aluminium and/or germanium allows smaller T–O–T angles, which promotes the construction of building units with small rings such as *s*4*r* and *d*4*r*. While boron, aluminium and germanium show preferences for building the *s*4*r* unit, germanium has advantages in forming the *d*4*r* unit^51^.

Therefore, a synthesis system that combines multiple framework T atoms ([Si,Ge,Al] or [Si,Ge,B]) and a simple OSDA (DMAP) was designed to synthesize zeolite materials. Such a system has received little attention because of the high risk of forming complex mixture products, which could be challenging to analyse and purify^52,75,76^. The simple OSDAs mainly play a pore-filling role in the crystallization of zeolites, which would allow different framework T atoms to direct the construction of diverse structure-building units and govern the formation of framework structures. The addition of aluminium or boron into the synthesis system of germanosilicate ([Si,Ge]) zeolites could reduce the influence of germanium, increase the stability of the materials, create more active sites and promote the formation of large or extra-large pore zeolites using simple OSDAs. The zeolite materials synthesized from the designed system would be promising for large-scale industrial productions and applications.

### Chemical reagents

The chemical reagents used in the synthesis experiments include Ludox (SiO_2_, HS-40, 40 wt%, Sigma-Aldrich), DMAP (98 wt%, TCI Shanghai), germanium oxide (GeO_2_, 99.0 wt%, Sinopharm Chemical Reagent), aluminium hydroxide (Al(OH)_3_, 99.0 wt%, Sinopharm Chemical Reagent), boric acid (H_3_BO_3_, 99.0 wt%, Sinopharm Chemical Reagent), hydrofluoric acid (HF, 40 wt% in water solution, Sinopharm Chemical Reagent) and distilled water.

### Synthesis of zeolite materials

In a typical synthesis procedure of the products prepared from [Si,Ge,Al] and [Si,Ge,B] systems, GeO_2_ and DMAP were first added into distilled water in a 23 ml Teflon liner, and the mixture was stirred at room temperature until GeO_2_ and DMAP were dissolved. Al(OH)_3_ or H_3_BO_3_ was then introduced under stirring. After Al(OH)_3_ or H_3_BO_3_ had dissolved, Ludox was added. HF solution was introduced dropwise under stirring after the gel became homogeneous. Then, the gel was aged at room temperature for 2 h under stirring. For the products synthesized from the [Si,Ge] system, only the step of introducing Al(OH)_3_ or H_3_BO_3_ was omitted. The gels with molar compositions presented in Table 1 were all crystallized at 170 °C for 10 days in 23 ml Teflon-lined stainless autoclaves under the static condition. Specifically, for the synthesis of product A, 0.523 g GeO_2_ and 3.665 g DMAP were first added into 3.600 g H_2_O, and 0.289 g Al(OH)_3_ was then introduced. Finally, 7.500 g Ludox and 1.501 g HF were added to form a gel with a molar composition of 1.0 SiO_2_ : 0.1 GeO_2_ : 0.0367 Al_2_O_3_ : 0.6 OSDA : 0.6 HF : 10 H_2_O (Si/Ge = 10, (Si + Ge)/Al = 15). For the synthesis of product B, a two-step heating programme (110 °C for 1 day and then 170 °C for 5 days) was applied for the crystallization, which involved mixing 1.046 g GeO_2_, 3.665 g DMAP, 3.600 g H_2_O, 0.378 g Al(OH)_3_, 7.500 g Ludox and 1.501 g HF. A gel with a molar composition of 1.0 SiO_2_ : 0.2 GeO_2_ : 0.048 Al_2_O_3_ : 0.6 OSDA : 0.6 HF : 10 H_2_O (Si/Ge = 5, (Si + Ge)/Al = 12.5) was prepared. After the crystallization, the autoclave was quenched in cold water. The solid products were recovered by centrifugation, washed with deionized water and then dried at 110 °C for 12 h. The OSDAs in the zeolite framework structures were removed by calcination in air at 600 °C for 5 h.

### SerialRED and cRED data collection

All SerialRED and cRED data and transmission electron microscopy (TEM) images were collected on a JEOL JEM2100 LaB_6_ transmission electron microscope at 200 kV with a Timepix hybrid pixel detector (Amsterdam Scientific Instruments). SerialRED experiments were performed using the software Instamatic^41,45^. For the sample preparation, the polycrystalline sample was first crushed in an agate mortar, suspended in ethanol (99.5 wt%) and then dispersed by ultrasonication. A drop of the suspension was transferred onto a TEM grid with a continuous carbon film (CF400-Cu-UL, 400 mesh, Electron Microscopy Sciences) for the electron diffraction data collection. During the SerialRED experiment, the typical set-up uses the second smallest condenser lens aperture (which is around 10 μm when projected on the sample plane) globally. A partially condensed parallel beam was used as a virtual ‘selected area’ aperture for the 3D ED data collection of every single crystal. An exposure time of 0.5 s was used for each diffraction pattern recording. After every 10 diffraction patterns, the diffraction pattern was defocused to enable tracking the crystal position inside the virtual ‘selected area’ aperture. The crystal tracking frequency is automatically changed to after every two diffraction patterns when the algorithm detects the crystal is close to the edge of the aperture, in order to perform ‘robust’ crystal tracking. Methods for automatic screening of suitable crystals, adjustment of eucentric height and online crystal tracking have been discussed in the previous papers^41,45^. cRED data were collected via the software Instamatic with human input at every experimental step from locating the crystal of interest to tracking the crystal during stage rotation. The 3D ED data processing was conducted by DIALS, REDp, XDS and edtools^60,62,77,78^.

### High-throughput phase analysis using SerialRED

The high-throughput phase analysis was carried out using the HCA algorithm implemented in the package edtools as described by Wang et al.^45,61^. The HCA can be performed via unit-cell-based clustering and/or intensity-based clustering. For phases with sufficient differences in their unit cell parameters (identified by DIALS or XDS)^60,62^, unit-cell-based clustering would be enough to assign the datasets in clusters corresponding to the different phases. In edtools, we modified the calculation of the Euclidean distance (equation (1)) and used it as the metric for the clustering.

1 $$d\left( {i,j} \right) = \sqrt {\begin{array}{l}{\Delta}a^2\left( {i,j} \right) + {\Delta}b^2\left( {i,j} \right) + {\Delta}c^2\left( {i,j} \right) + {\Delta}\left( {\sin\alpha } \right)^2\left( {i,j} \right)\\\ \ + {\Delta}\left( {\sin\beta } \right)^2\left( {i,j} \right) + {\Delta}\left( {\sin\gamma } \right)^2\left( {i,j} \right)\end{array}}$$

In equation (1), we converted the angles into sine terms because of the ambiguity in the choice of the directions of the unit cell vectors from indexing (for example, an angle can be either 60° or 120° from the same unit cell). This distance metric is derived from the metric linear cell variation^79^. In this equation, *a*_*i*_, *b*_*i*_, *c*_*i*_, *α*_*i*_, *β*_*i*_ and *γ*_*i*_ correspond to the unit cell parameters from the *i*th dataset, where *a*, *b*, *c* are the unit cell lengths and *α*, *β*, *γ* are the unit cell angles. *a*_*j*_, *b*_*j*_, *c*_*j*_, *α*_*j*_, *β*_*j*_ and *γ*_*j*_ are the parameters from the *j*th dataset. Δa^2^(*i*, *j*) is defined as (*a*_*i*_ - *a*_*j*_)^2^. The same applies to Δb^2^(*i*, *j*) and Δc^2^(*i*, *j*). Δ(sin *α*)^2^(*i*, *j*) is defined as (sin *α*_*i*_ - sin *α*_*j*_)^2^. The same applies to Δ(sin *β*)^2^(*i*, *j*) and Δ(sin *γ*)^2^(*i*, *j*). The datasets are grouped based on the distance metric; each cluster includes datasets with similar unit cell parameters that are regarded as the same phase. Further improvement of the clustering can be achieved by introduction of different weighting schemes for the unit cell lengths and angles.

It is worth mentioning that the unit cell parameters determined from the SerialRED datasets may have large errors because of optical distortions of the microscope, the instability of the goniometer and inaccurate camera lengths which are strongly correlated to the unit cell dimensions. In addition, small rotation angles of the datasets could also lead to large errors in the unit cell determinations. Therefore, it is important to calibrate the camera lengths and collect the datasets under the same optical conditions near the eucentric height. A stable goniometer stage can also help to reduce the errors of the unit cell parameters determined from our SerialRED data, as it would be easier to collect datasets with large rotation ranges. During our data processing using both DIALS and XDS, a fixed camera length was used for all the SerialRED datasets without further refinement.

For phases with similar unit cell parameters, one can further apply the intensity-based clustering, which is based on the Pearson correlation coefficient calculated from the common reflections in two datasets (calculated by XSCALE) as the distance metric (equation (2))^61,62^.

2 $$d\left( {i,j} \right) = \sqrt {1 - {\mathrm{CC}}_{\mathrm{I}}^2\left( {i,j} \right)}$$

The CC_I_ value in equation (2) represents the Pearson correlation between datasets *i* and *j* calculated from the intensities of the common reflections in the datasets. Phase analysis using intensity-based clustering works best when there are enough common reflections in the datasets, which requires the datasets have high enough completeness.

Notably, for crystals with similar unit cell parameters (for example, *b* and *c* have similar values), there might be a risk that unit-cell-based clustering misclassifies the phases and introduces ambiguity in the reflection indexing. Consequently, the intensity-based clustering may fail. A similar indexing ambiguity problem has been resolved for serial crystallography data with single-shot frames^80^, which has been implemented in DIALS (dials.cosym)^81^. We will keep investigating the potential of intensity-based clustering of 3D ED datasets for tackling such a problem. In this work, intensity-based clustering was only used to identify and merge the highly correlated 3D ED datasets for structure determination. Because of the relatively low completeness due to rotation range of the current SerialRED data, we did not use intensity-based clustering for phase analysis.

The structure solution was performed using direct methods in SHELXT^64^. The kinematic structure refinement was conducted using SHELXL^82^ and Shelxle^83^. Atomic scattering factors of electrons were used^84^.

### SPXRD and other general characterizations

The SPXRD data were collected in a 0.5 mm capillary on the BL14B1 X-ray diffraction beamline at the Shanghai Synchrotron Radiation Facility using a wavelength of 0.68950 Å. The SPXRD patterns were collected in the 2*θ* range 2.500°–38.000° with 0.004° data binning. In-house PXRD patterns were recorded on a D8 Advance X-ray diffractometer using Cu Kα radiation, operating at 40 kV and 40 mA. SEM images were taken on a field emission XL30E scanning electron microscope (FEI). Surface areas and pore volumes were obtained from nitrogen adsorption/desorption isotherms using multipoint Brunauer–Emmett–Teller and *t*-plot methods. The experiments were performed on Micromeretic ASAP2020M physisorption apparatus at a liquid nitrogen temperature of −196 °C. The amounts of Si, Al, B and Ge were quantified by inductively coupled plasma on a Varian 725-ES instrument after dissolving the samples in HF solution. ^29^Si, ^27^Al, ^13^C and ^19^F solid-state magic-angle-spinning NMR spectra were recorded on a Varian VNMRS-400WB spectrometer. ^29^Si NMR spectra were acquired with a 7.5 mm T3HX probe at 79.43 MHz and a spinning rate of 3 kHz. ^27^Al spectra were recorded at a frequency of 104.18 MHz, a spinning rate of 10.0 kHz and a recycling delay of 4 s. ^13^C NMR spectra were recorded with a 7.5 mm T3HX probe at 100.54 MHz and a spinning rate of 5 kHz. ^19^F solid-state NMR was recorded on a Bruker AVANCEIII 500WB spectrometer with a 2.5 mm probe at 376.5 MHz with a spinning rate of 30 kHz. The concentration of Lewis and Brønsted acid sites was determined after the adsorption of pyridine by Fourier transform infrared spectroscopy on a Thermo Fisher Nicolet 380 spectrometer using the sample wafer. Temperature programmed desorption using NH_3_ was carried out on TPD/TPR Altamira AMI-3300 equipment.

### Catalytic tests and performances

Product B catalyst was obtained directly via calcination of its as-made product in air at 600 °C for 5 h. As-made **MOR** product was purchased from Shanghai Novel Chemical Technology. As-made **SFE** product was prepared from a gel with a molar composition of 1 SiO_2_ : 0.05 B_2_O_3_ : 0.01 Al_2_O_3_ : 0.6 DMAP : 25 H_2_O with the assistance of **SFE** type borosilicate seed (5 wt%) as previously reported^70^. The gel was crystallized in 100 ml Teflon-lined stainless autoclaves at 170 °C for 3 days under rotation (20 r.p.m.). In this synthesis process, 10.995 g DMAP was first dissolved in 54.000 g H_2_O, 0.939 g H_3_BO_3_ was then added and the mixture was stirred at room temperature for 30 min. Then, 0.355 g NaAlO_2_ (43 wt% Al_2_O_3_, 35 wt% Na_2_O, Zibo Lier Chemical) was added. After that, 22.5 g Ludox was added dropwise. Finally, 0.45 g seed was added. The seed was prepared by heating a gel with a molar composition of 1 SiO_2_ : 0.05 B_2_O_3_ : 0.6 DMAP : 25 H_2_O at 170 °C for 3 days under rotation (20 r.p.m.). The synthesis procedures of the seed are almost the same as those of the as-made **SFE** product, except for the adding of NaAlO_2_ and the seed.^70^. To make **MOR** and **SFE** into acid catalysts, their as-made products were first calcined in air at 600 °C for 5 h. Then, they were converted into the acid forms via ion-exchange using ammonium nitrate (10 wt% aqueous solution) with a solid-to-liquid weight ratio of 1:30 at 60 °C for 1 h. This ion-exchange procedure was repeated three times. The **MOR** and **SFE** solid powder catalysts were obtained after separation, drying (in air, 110 °C, 12 h) and calcination (in air, 600 °C, 5 h).

The catalytic performance of product B was tested on the disproportionation of IPN(97.6 wt% (65.1 wt% 2-IPN in IPN), purchased from China Medicine Group) and compared with those of the **MOR** and **SFE** catalysts. Disproportionation of IPN is a complex reaction that produces numerous diisopropylnaphthalene (DIPN) isomers, such as 1,3-DIPN, 1,7-DIPN, 2,6-DIPN (target product) and 2,7-DIPN. The 2,6-DIPN/2,7-DIPN ratio in the product is highly related to the pore architecture of zeolite catalysts^67,85^. Disproportionation reactions were conducted in a 100 ml stainless steel autoclave with magnetic agitation under the following reaction conditions: 35 g IPN : 1.5 g catalyst, autogenous pressure, 250 °C and a reaction time of 6 h. After the reaction was finished, the autoclave was cooled down to room temperature with water, and the catalysts were filtrated. The liquid reaction products were analysed in an Agilent 7890A gas chromatograph equipped with an Agilent 19091N-236 HP-INNOWax capillary column (60 m × 250 μm × 0.5 μm) by using a flame ionization detector.

## Online content

Any methods, additional references, Nature Portfolio reporting summaries, source data, extended data, supplementary information, acknowledgements, peer review information; details of author contributions and competing interests; and statements of data and code availability are available at 10.1038/s41557-022-01131-8.

## Supplementary information

Supplementary Information Supplementary Figs. 1–23, Tables 1–10, notes and references.

Supplementary Data 1 Raw data for Supplementary Fig. 8.

Supplementary Data 1 Raw data for Supplementary Fig. 12.

Supplementary Data 1 Raw data for Supplementary Fig. 20.

Supplementary Data 1 Crystallographic data for the zeolite phases in product A determined by SerialRED.

Supplementary Data 1 Crystallographic data for the zeolite phases in product B determined by SerialRED.

Supplementary Data 1 Crystallographic data for the zeolite phases in Product B determined by SPXRD.

## Data Availability

All the data that support the findings of this study are available in this paper and its Supplementary Information. The raw SerialRED data of this study are available at 10.5281/zenodo.5728145^86^. Source data for Fig. 2 and Supplementary Figs. 8, 12 and 20 are provided with this paper. Source data are provided with this paper.

## Source data

Source Data Fig. 2 PXRD patterns of product A and product B.

## Code availability

The Instamatic (v.1.1.0) Python package for SerialRED experiments is available at 10.5281/zenodo.3470096 or https://github.com/instamatic-dev/instamatic under the terms of GNU General Public License v.3.0. The edtools (v.1.0.4) Python package for SerialRED data processing is available at 10.5281/zenodo.6952810 or https://github.com/instamatic-dev/edtools under the terms of BSD 3-Clause ‘New’ or ‘Revised’ License. The installation and documentation information for the Instamatic and edtools packages can be found in their readme.md files. DIALS (v.1.10.0) for on-the-fly indexing of SerialRED datasets is available at https://github.com/dials/dials/releases/tag/v1.10.0 under the terms of BSD 3-Clause ‘New’ or ‘Revised’ License. XDS (v. 10 January 2022, BUILT = 20220220) for reduction of SerialRED datasets is available at https://xds.mr.mpg.de/html_doc/downloading.html. The guidelines for the installation of the XDS package can be found at https://strucbio.biologie.uni-konstanz.de/xdswiki/index.php/Installation. A step-by-step demo of SerialRED data processing using edtools is provided in the Supplementary Notes, as well as at https://edtools.readthedocs.io/en/latest/examples/edtools_demo.html.
