# Supplementary material for: High-throughput phase elucidation of polycrystalline materials using serial rotation electron diffraction
Source: Nat Chem. 2023 Jan 30;15(4):483–90. doi: 10.1038/s41557-022-01131-8 (PMC10070184; doi:10.1038/s41557-022-01131-8)
Supplement: Supplementary file 1 — Supplementary Figs. 1–23, Tables 1–10, notes and references. [file 41557_2022_1131_MOESM1_ESM.pdf]

nature chemistry

Article https://doi.org/10.1038/s41557-022-01131-8

High-throughput phase elucidation of polycrystalline materials using serial rotation electron diffraction

In the format provided by the authors and unedited

Supplementary materials for

High-throughput Phase Elucidation of Polycrystalline Materials Using Serial Rotation Electron Diffraction

Yi Luo,[1,2,5] Bin Wang,[1,5] Stef Smeets,[3] Junliang Sun,[4] Weimin Yang,[2] and Xiaodong Zou[1]

[1]Department of Materials and Environmental Chemistry, Stockholm University, SE-106 91 Stockholm, Sweden.

[2]State Key Laboratory of Green Chemical Engineering and Industrial Catalysis, Sinopec Shanghai Research Institute of Petrochemical Technology, 1658 Pudong Beilu, Shanghai 201208, China.

[3]Netherlands eScience Center, Science Park 140, 1098 XG Amsterdam, The Netherlands.

[4]College of Chemistry and Molecular Engineering, Beijing National Laboratory for Molecular Sciences, Peking University, Beijing 100871, China.

[5]Y. Luo and B. Wang contributed equally to this work

Corresponding authors: Xiaodong Zou (xzou@mmk.su.se)

Weimin Yang (yangwm.sshy@sinopec.com).

Table of contents

Supplementary Fig. 1……………………………………………………….………………………...3
Supplementary Fig. 2…………………………………………………………….………………...4
Supplementary Fig. 3……………………………………….…………………………………...5
Supplementary Fig. 4……………………………………..……………………………………..6
Supplementary Fig. 5……………………………………………………………………….……...7
Supplementary Fig. 6……………………………………………………………………………...8
Supplementary Fig. 7………………………………………………………………………..…...9
Supplementary Fig. 8…………………………………………………………………………10
Supplementary Fig. 9…………………………………………………………….……………11
Supplementary Fig. 10……………………………………………………………….………...12
Supplementary Fig. 11…………………………………………………………………….……...13
Supplementary Fig. 12……………………………………………………………….…………...14
Supplementary Fig. 13……………………………………………………………………………...15
Supplementary Fig. 14……………………………………………………………….…………...16
Supplementary Fig. 15…………………………………………………………………….……...17
Supplementary Fig. 16…………………………………………………………………….……...18
Supplementary Fig. 17……………………………………………….…………………………...19
Supplementary Fig. 18……………………………………………………….……………………...20
Supplementary Fig. 19……………………………………………………….…………………...21
Supplementary Fig. 20……………………………………………………………….………...22
Supplementary Fig..21…………………………………………………………………………...23
Supplementary Fig. 22……………………………………………………………………………...24
Supplementary Fig. 23……………………………………………………………………………...25
Supplementary Table 1……………………………………………………….……………...26
Supplementary Table 2……………………………………………………….……………...28
Supplementary Table 3…………………………………………………………………….....29
Supplementary Table 4……………………………………………………….…………….....30
Supplementary Table 5……………………………………………………………….………...32
Supplementary Table 6……………………………………………………….…………...33
Supplementary Table 7…………………………………………………………………….....34
Supplementary Table 8……………………………………………………………………35
Supplementary Table 9………………………………………………………………….....36
Supplementary Table

10………………………..…………………………………………………....37
Supplementary notes………………………………………………………………………...38
References…………………………………………………………………………………...41

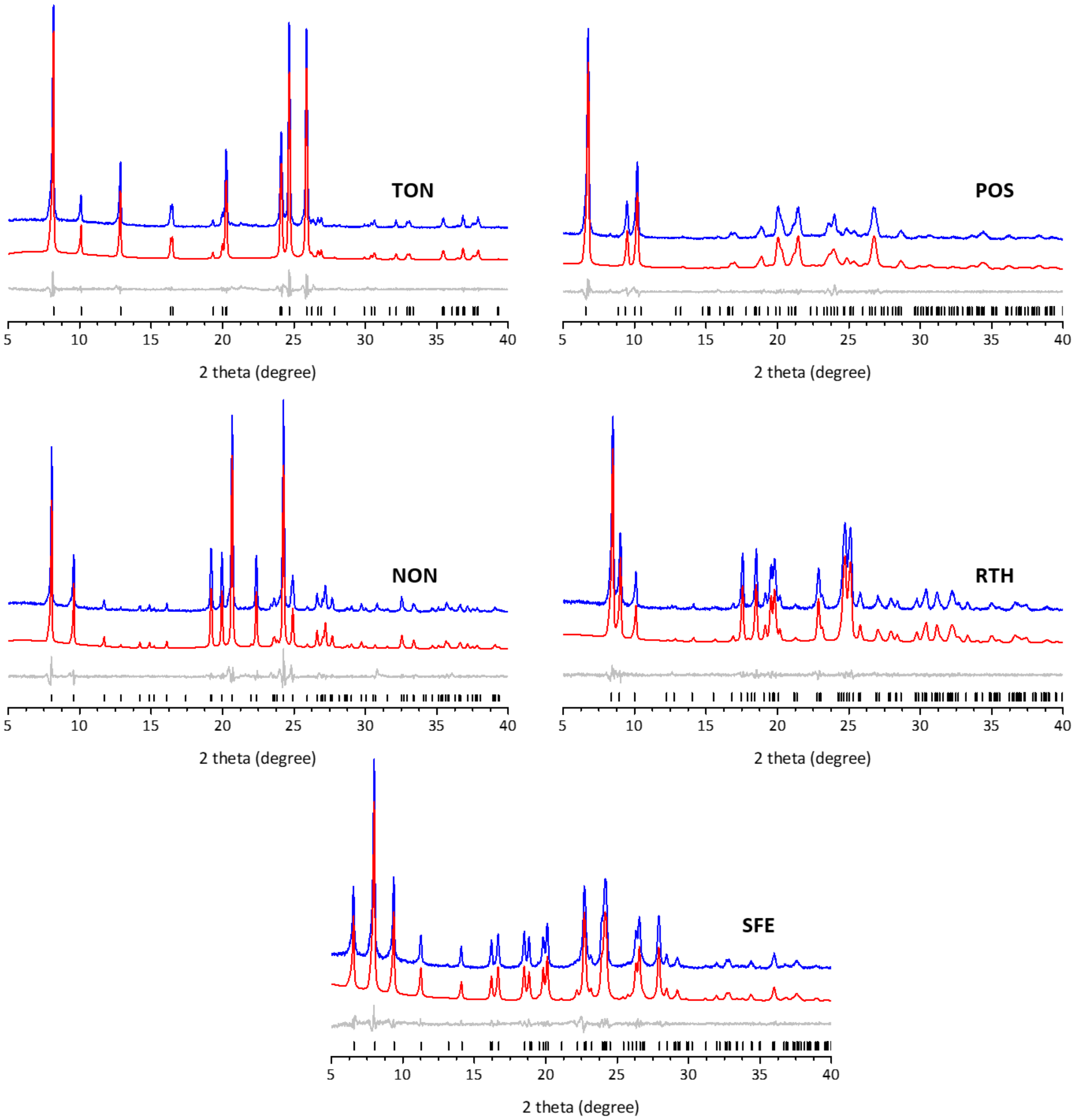

Supplementary Fig. 1 Pawley fit profiles of the PXRD patterns (Cu Kα) of pure zeolite phases. The pure zeolite phases were obtained in exploring our designed synthesis system and identified by PXRD. Observed (blue line), calculated (red line), as well as difference (gray line) profiles are presented. The black tick marks under the patterns are the positions of the Bragg reflections.

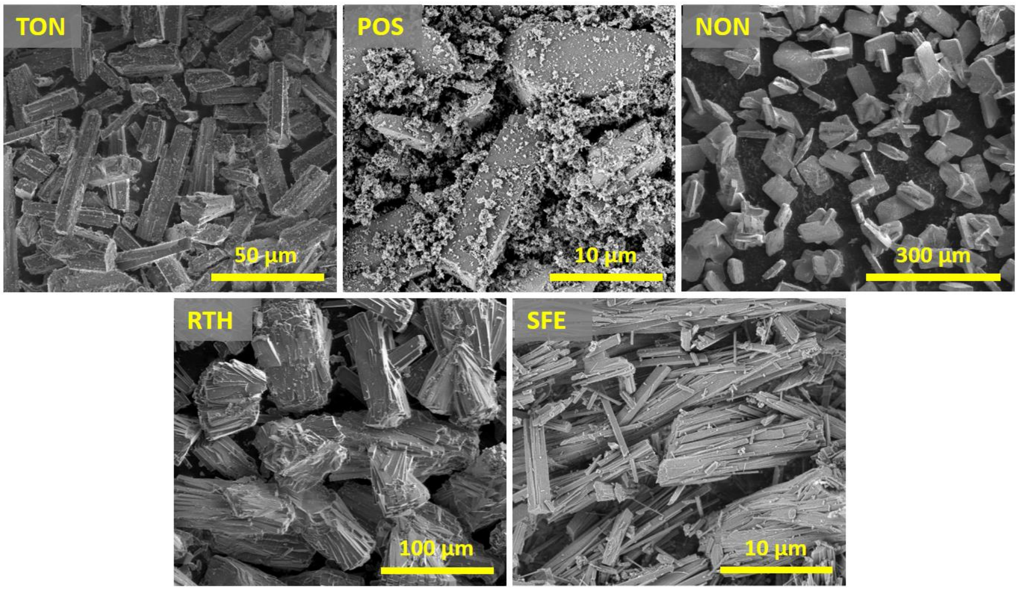

Supplementary Fig. 2 SEM images of the pure zeolite phases. The products were prepared from the designed synthesis system that combines multiple framework T atoms ([Si,Ge], [Si,Ge,Al] or [Si,Ge,B]) and a simple pore-filing OSDA (DMAP). While **TON**, **POS**, **RTH** and **SFE** show needle-like morphologies, and **NON** has a cuboidal morphology. Some small amorphous particles are presented on the **POS** crystals.

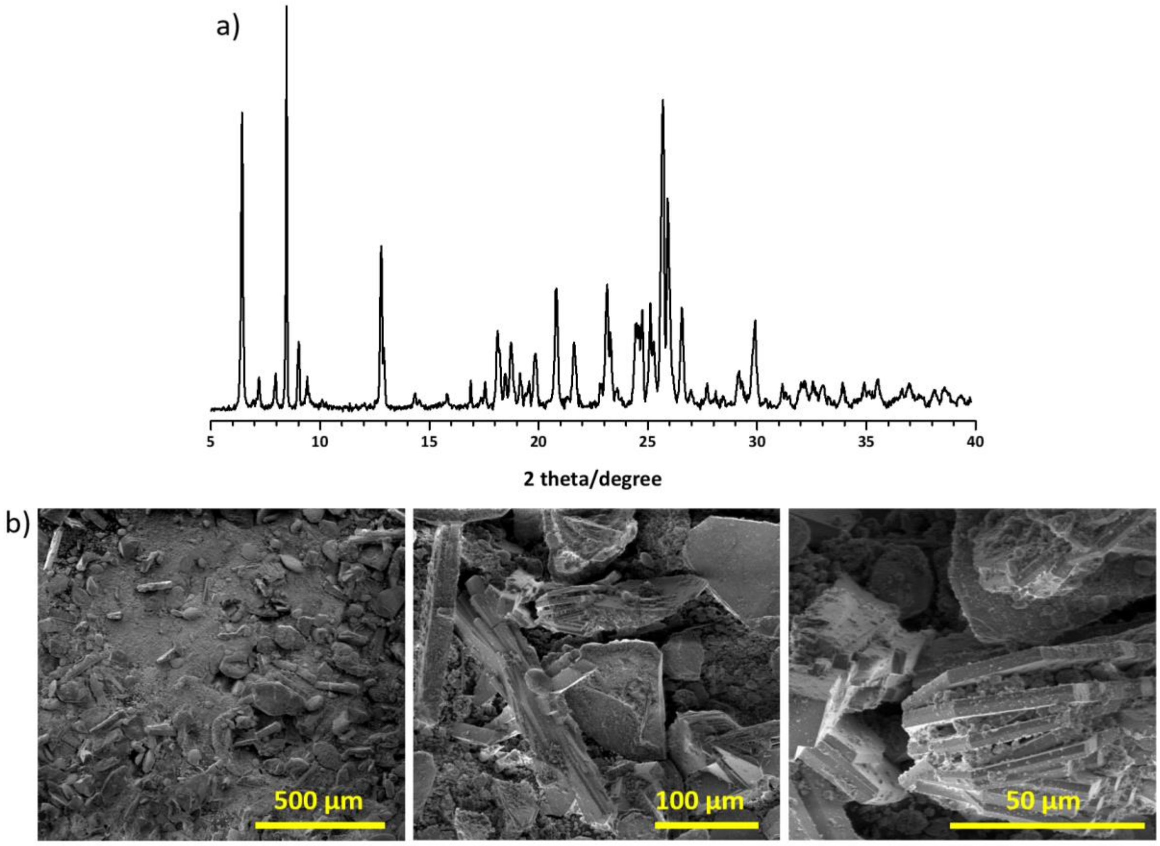

Supplementary Fig. 3 PXRD pattern (a) and SEM images (b) of Product A. Crystals with needle- and plate-like morphologies were observed. The PXRD pattern and one of the SEM images are also presented in Fig 2 of the main text.

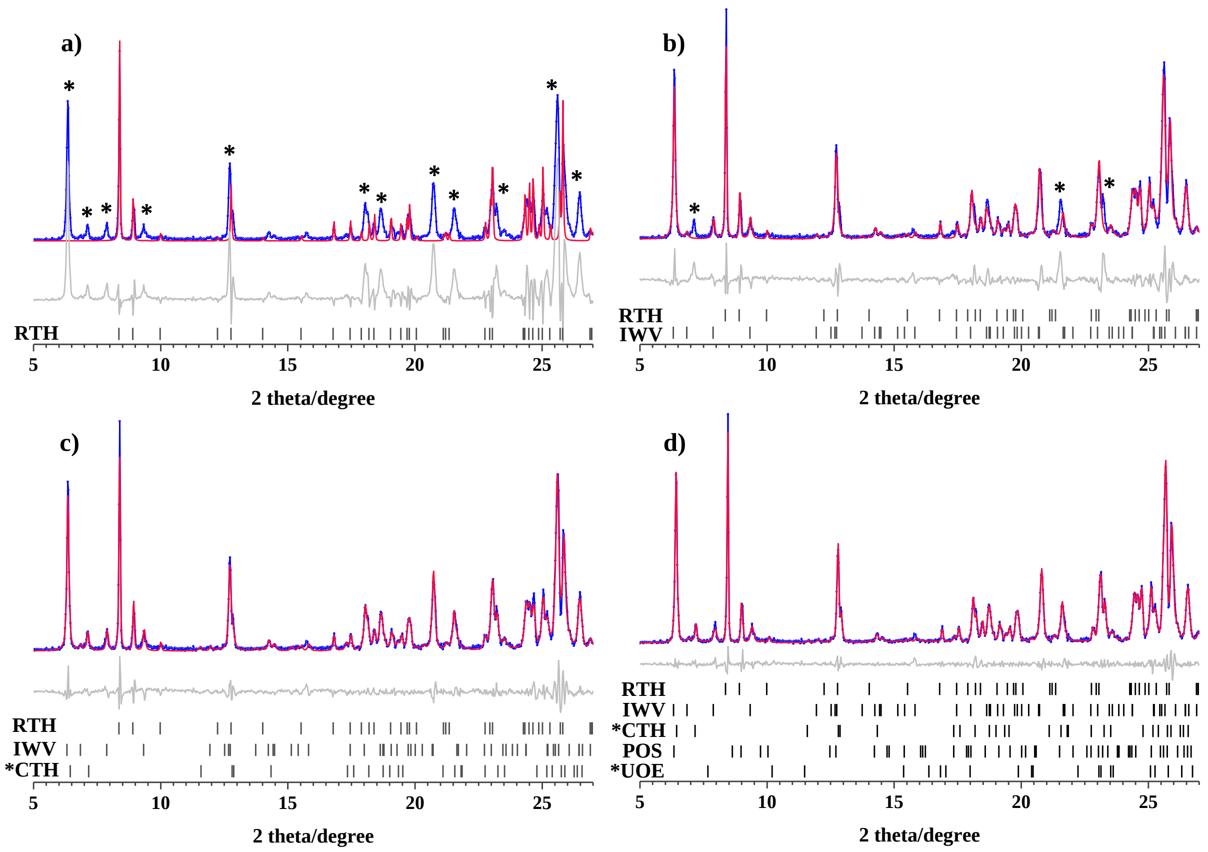

Supplementary Fig. 4 Pawley fit profiles of the PXRD pattern (Cu Kα) of Product A with a different number of phases. a) **RTH**, b) **RTH** and **IWV**, c) **RTH**, **IWV** and ***CTH**, and d) **RTH**, **IWV**, ***CTH**, **POS** and ***UOE**. The Pawley fit results indicate that **RTH**, **IWV** and ***CTH** are the major phases, and ***UOE** and **POS** are the minor phases. Without the aid of conventional 3D ED, only **RTH** could be identified by PXRD. With conventional 3D ED, two of the major phases **RTH** and **IWV** were found, but the third phase ***CTH** was missed. The minor phases **POS** and ***UOE** could hardly be detected by PXRD, and also did not found by conventional 3D ED. All the five phases were reliably identified using SerialRED. Observed (blue dots), calculated (red line), as well as difference (grey line) profiles are presented. The tick marks are the positions of the Bragg reflections belonging to each phase. Unindexed peaks were marked by asterisks.

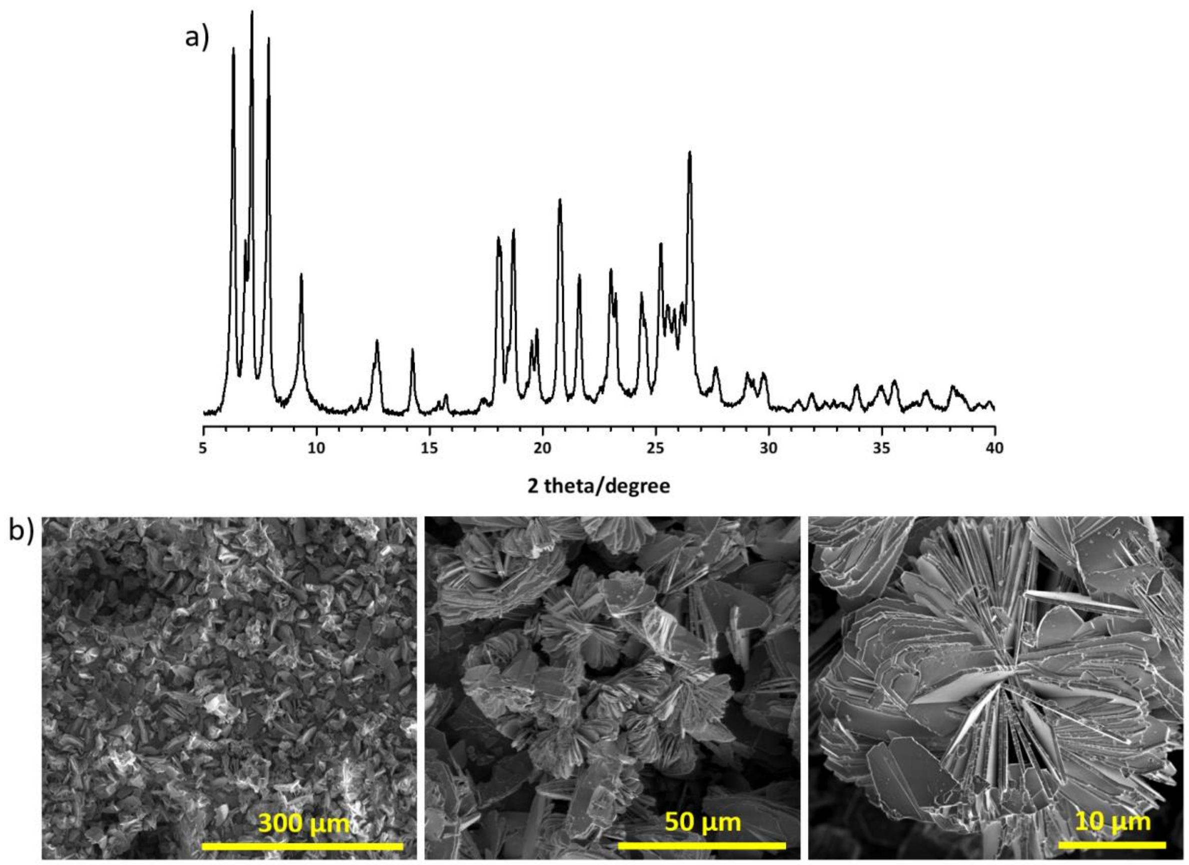

Supplementary Fig. 5 PXRD pattern (a) and SEM images (b) of Product B. Only the high-quality crystals with uniform plate-like morphology were observed, which could lead to misinterpretation that Product B was phase pure. The PXRD pattern and one of the SEM images are also presented in Fig 2 of the main text.

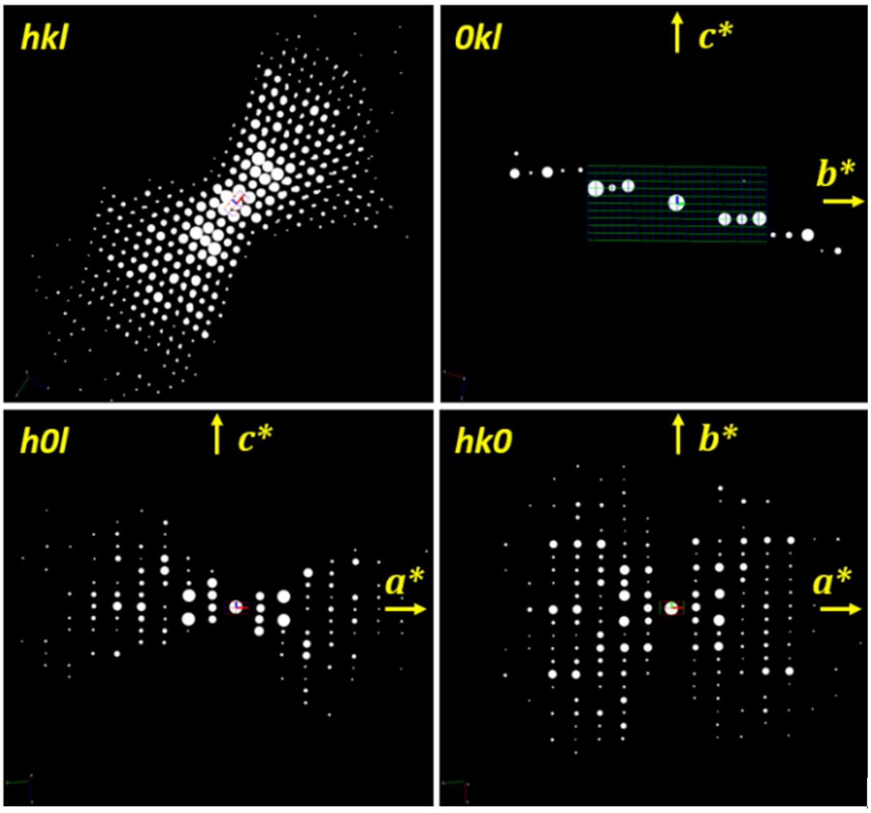

Supplementary Fig. 6 Reciprocal lattice of one plate-like crystal in Product B reconstructed from the conventional 3D ED data. The reflection conditions deduced from the 3D reciprocal lattice and the three 2D slices are *hkl*: *h*+*k*=2n, *h*+*l*=2n, *k*+*l*=2n, 0*kl*: *k*=2n, *l*=2n, *h*0*l*: *h*=2n, *l*=2n, *hk*0: *h*=2n, *k*=2n, *h*00: *h*=2n, 0*k*0: *k*=2n, 00*l*: *l*=2n. An orthorhombic unit cell (a= 14.34Å, b= 26.11Å, c=28.81Å) with possible space groups of *Fmmm*, *Fmm*2, and *F*222 was elucidated, which is in good agreement with that of **IWV** zeolite. The structure model from the 3D ED data further confirmed that Product B contains a zeolite phase with an **IWV** type framework structure.

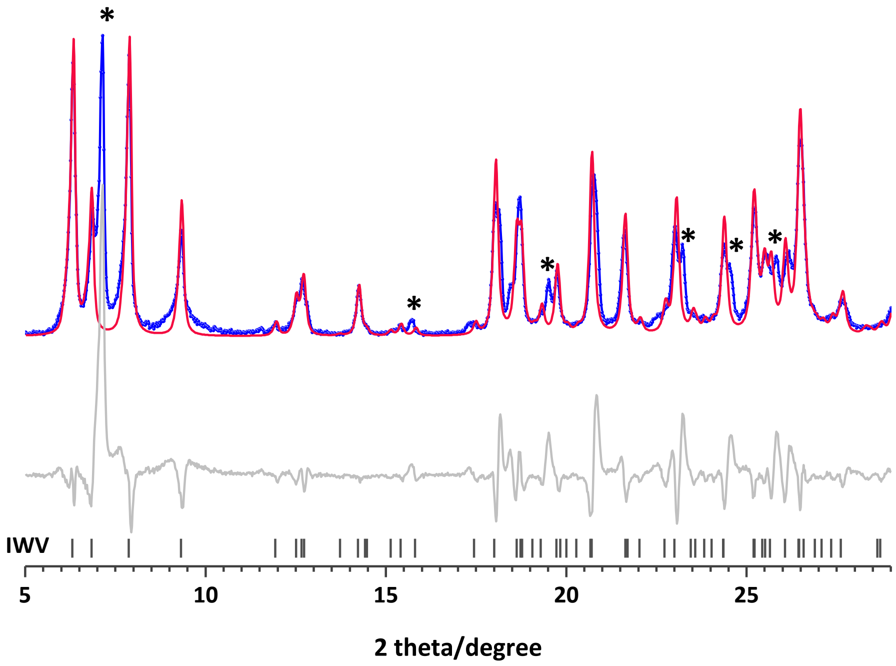

Supplementary Fig. 7 Pawley fit profiles of the PXRD pattern (Cu Kα) of Product B with only one phase IWV. Observed (blue dots), calculated (red line), as well as difference (grey line) profiles are presented. The difference profile shows that some strong reflections were not indexed (marked by asterisks). This indicates that Product B may contain other phases. Conventional 3D ED failed to identify it.

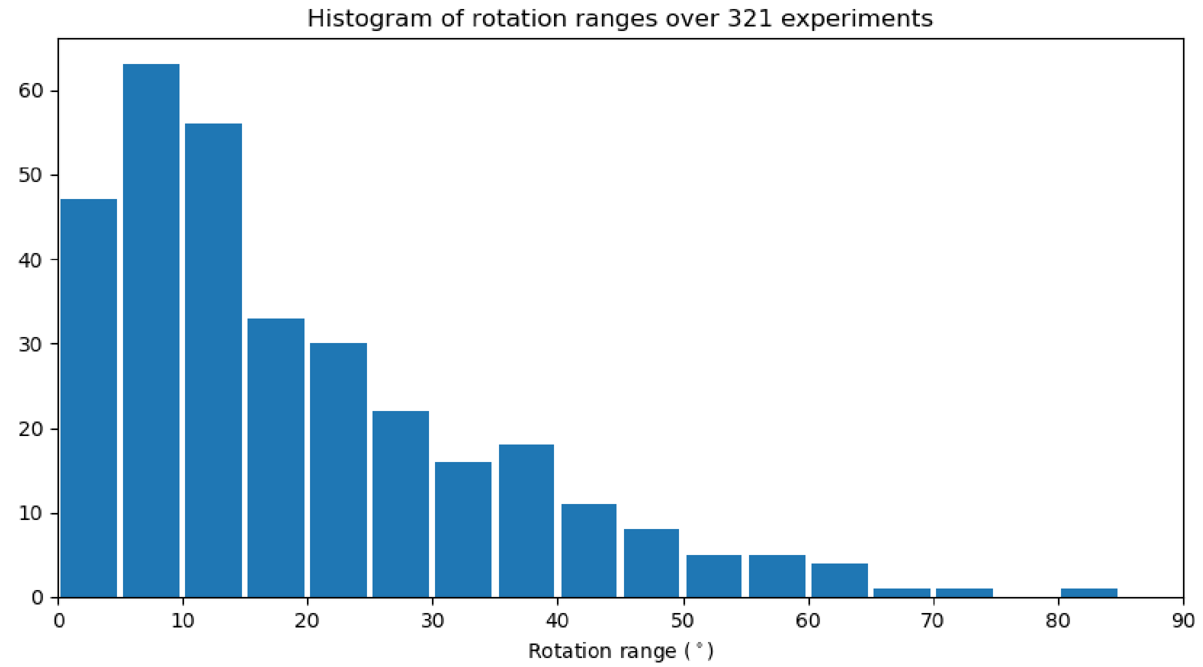

Supplementary Fig. 8. Rotation range histogram for the SerialRED experiment on Product A. 321 datasets were collected within a 6 hours' runtime. Source data are available in the additional supplementary files.

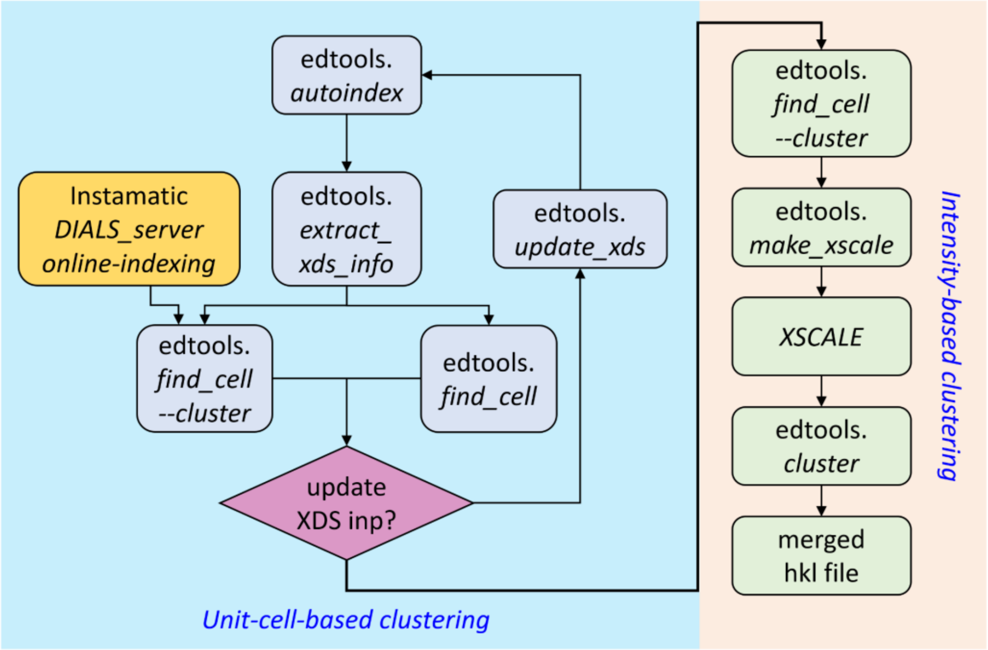

Supplementary Fig. 9 Flowchart of the processing and hierarchical clustering analysis (HCA) of SerialRED data by *edtools*. The HCA can be performed via unit-cell-based clustering and intensity-based clustering. For datasets with sufficiently different unit cell parameters (identified by *DIALS* or *XDS*), unit-cell-based clustering tells them apart by applying the modified Euclidean distance (Eq. 1 in the Methods section) as the metric. For datasets with similar or the same unit cell parameters, they can be separated effectively using a second step intensity-based clustering, taking the Pearson correlation coefficient between symmetry-related reflections in two datasets (calculated by *XSCALE*) as the distance metric. Datasets grouped after the two clustering steps will be automatically merged using *XSCALE*, and the obtained intensity files can be directly used for structure solution and refinement.

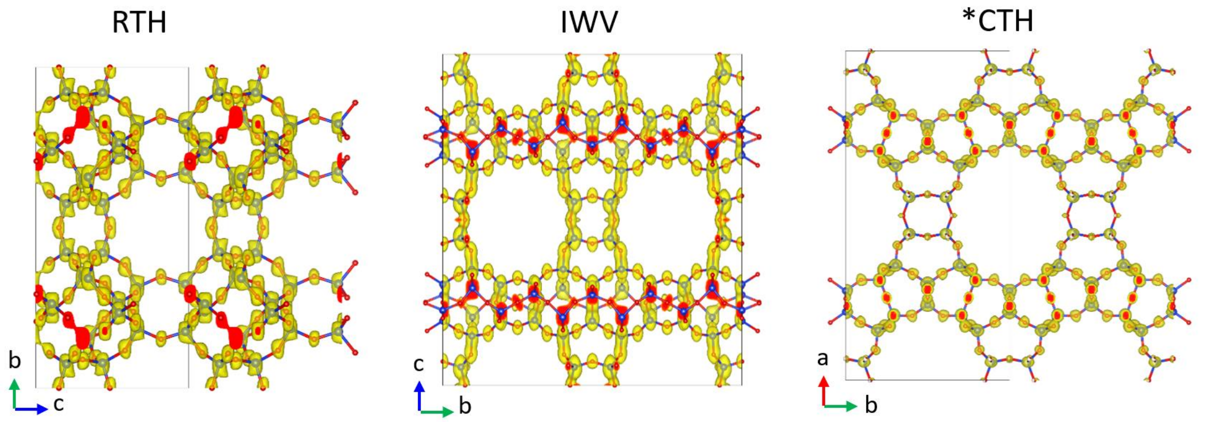

Supplementary Fig. 10 Framework structures and corresponding electrostatic potential maps of the major phases RTH, IWV and *CTH in Product A determined from the merged SerialRED data. This demonstrates that SerialRED is not only a powerful method in phase analysis but also can be used for ab initio structure determination. All the atoms in these three framework structures could be directly located, including those in ***CTH** that has a complex framework structure with disorders.

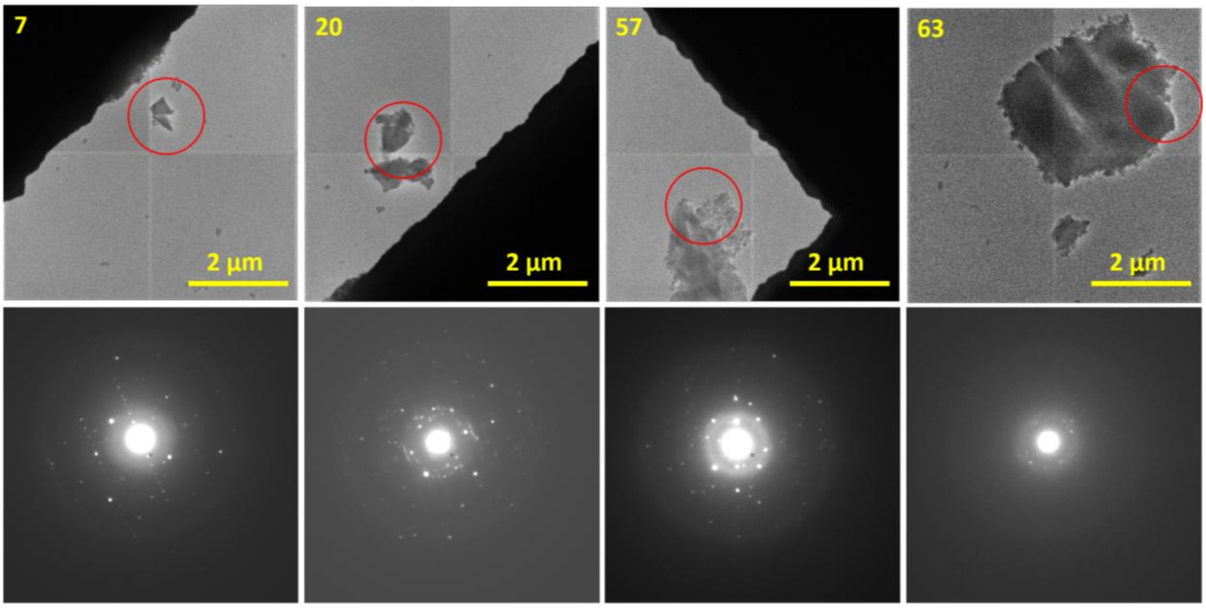

Supplementary Fig. 11 TEM images (up) and corresponding diffraction patterns (down) of selected crystals with their unit cells unclassified in Fig. 3 and Supplementary Table 1. The number in yellow at the upper left corner of each image is the dataset index in Fig. 3 and Supplementary Table 1. The unclassified unit cells mainly associate to datasets containing multiple lattices (7, 20, 57) or with poor quality (63), resulting in unit cell parameters with large errors or being completely wrong.

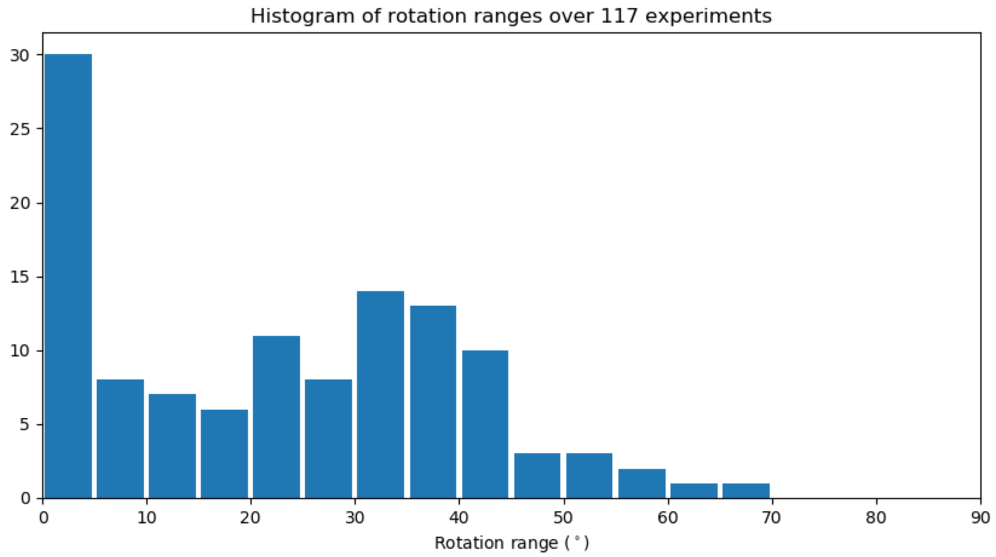

Supplementary Fig. 12 Rotation range histogram for the SerialRED experiment on Product B. 117 datasets were collected within a 4 hours' runtime. Source data are available in the additional supplementary files.

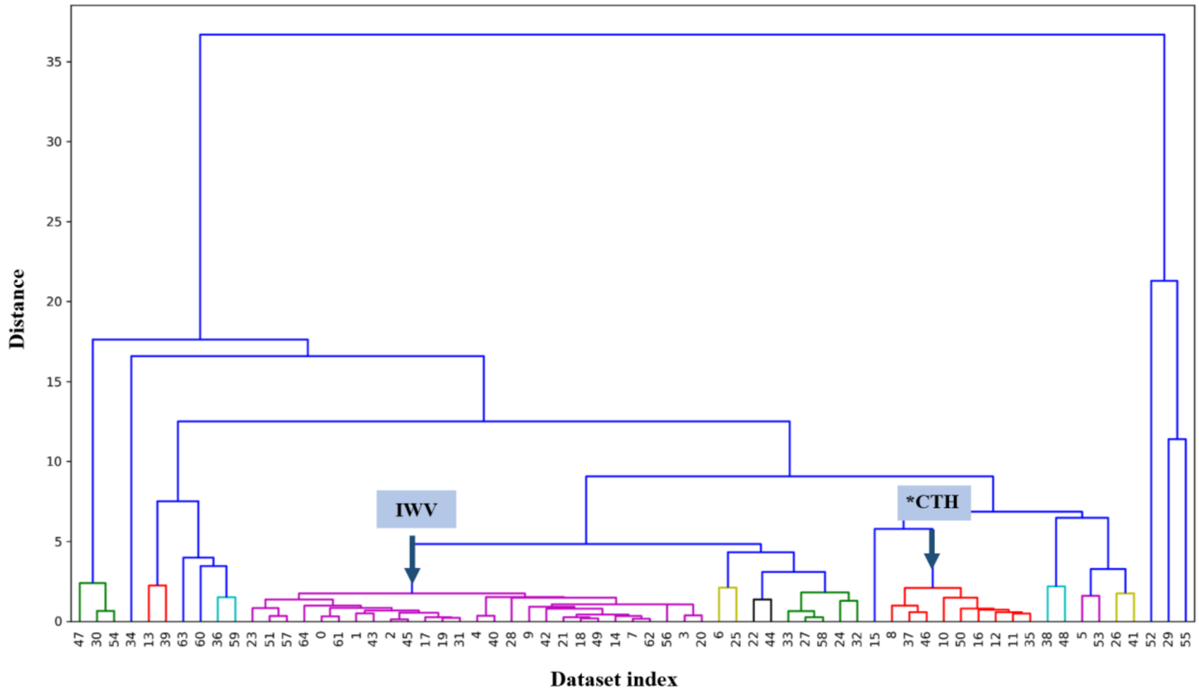

Supplementary Fig. 13 Dendrogram showing the results of the hierarchical cluster analysis (HCA) of Product B. HCA on preliminary indexing results of 65 datasets (with rotation ranges larger than 20˚) from Product B parsed from XPARM.XDS files from *XDS* processing. In total, 117 datasets were collected during a 4-hour' automated run of SerialRED, 89 of them could be indexed and return unit cells. Among them, 65 had rotation ranges larger than 20˚ and were used. The remaining 24 were discarded because of the risk of large errors in the unit cell determination. HCA has shown two major zeolite phases **IWV** (27 datasets) and ***CTH** (9 datasets), when setting the Euclidean distance cut at 2.48. The unclassified datasets can be attributed to the multi-lattices and/or poor data quality, which may result in incorrect unit cell parameters. In Product B, both **IWV** and ***CTH** have the same plate-like crystal morphology. Many of them are aggregated together, resulting in considerable SerialRED datasets from multi-crystals. The unit cell parameters from all 65 datasets for HCA are shown in Supplementary Table 4. Here we opted to use preliminary unit cell determination results from XPARM.XDS because the clustering results are clearer compared to that from the *DIALS* server.

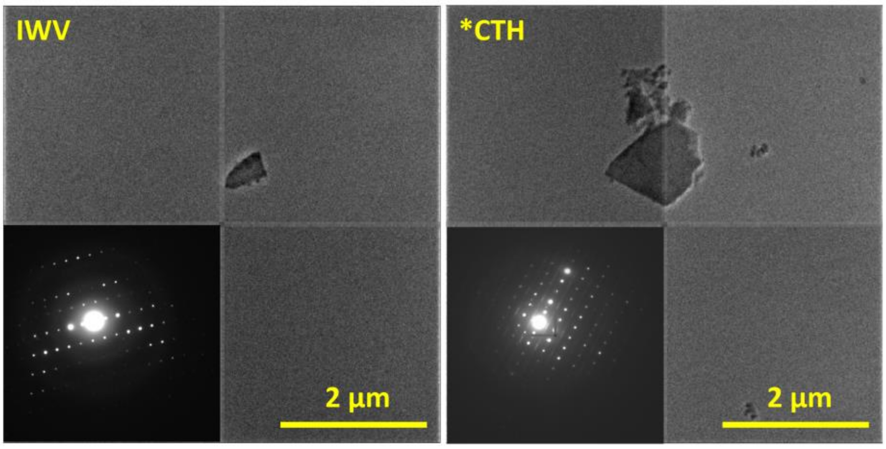

Supplementary Fig. 14 TEM images and the corresponding electron diffraction patterns of Product B. The typical morphologies and corresponding electron diffraction patterns (bottom left) of the crushed crystals of **IWV** (left) and ***CTH** (right).

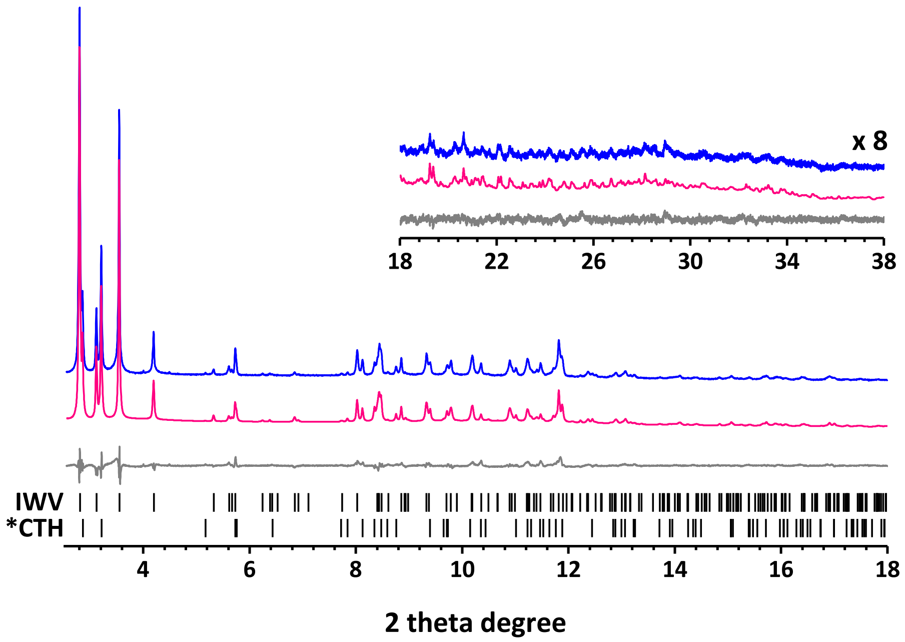

Supplementary Fig. 15 Profiles of Rietveld refinement of Product B (calcined) against the SPXRD data (wavelength: 0.6895 Å). Observed (blue line), calculated (pink line), as well as difference (gray line) profiles are presented. The profiles in the inset are magnified by eight times to show more details in the high-angle range. The black tick marks below the patterns are the positions of the Bragg reflections belonging to **IWV** and ***CTH**. The refined phase composition is 67% **IWV**: 33% ***CTH**. Due to the peak overlapping, it was not possible to identify the phase ***CTH** by PXRD.

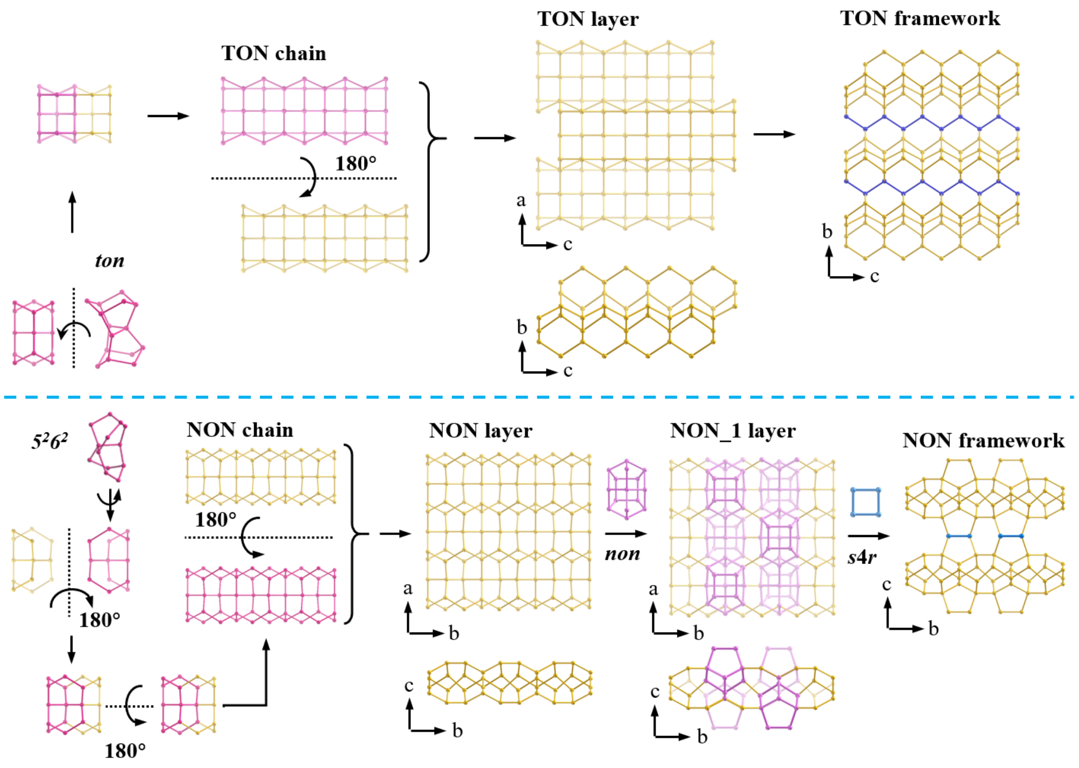

Supplementary Fig. 16 Structure relationship between the TON and NON frameworks. The two frameworks contain very similar layers (**TON** layer and **NON** layer) that are constructed from very similar building units (*ton*, $5^2 6^2$). The **TON** framework can be directly built from the **TON** layers, while **NON** framework can be built by the **NON_1** layers, which are constructed after embedding *non* units on the **NON** layers. The formation of the **NON** (Si/Ge=15, (Si+Ge)/T^{III}=30~100) type structure was realized by introducing a small amount of Al or B into the synthesis system of **TON** (Si/Ge=15, (Si+Ge)/T^{III}=∞), which indicates Al or B triggers the formation of the *non* building units. O atoms have been omitted in the structures for clarity.

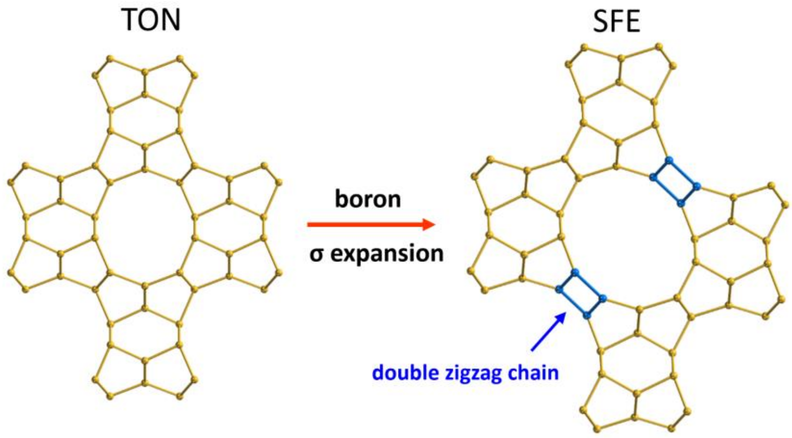

Supplementary Fig. 17 Structure relationship between the TON and SFE frameworks.The **SFE** type framework could be constructed from that of **TON** through σ expansion[1]. The σ expansion and formation of **SFE** (Si/Ge=15, (Si+Ge)/B=5-25) could be achieved by introducing a significant amount of B into the synthesis system of **TON** (Si/Ge=15, (Si+Ge)/B=∞), which shows B has the advantage in promoting the formation of small *s*4*r* units and building the double zigzag chains[1]. O atoms have been omitted in the structures for clarity.

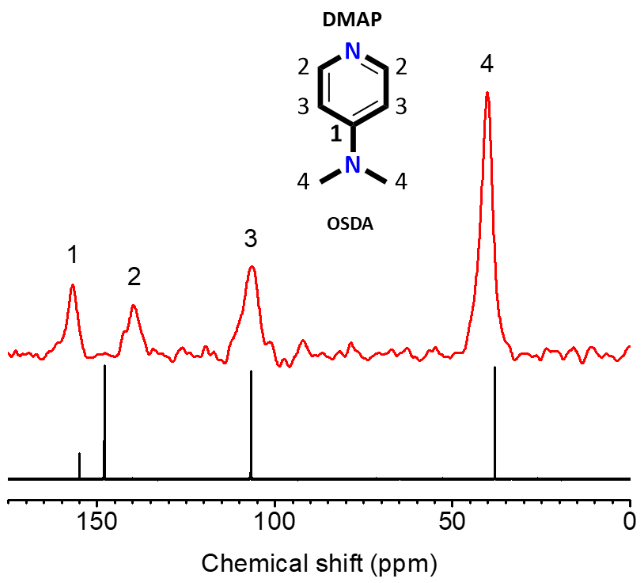

Supplementary Fig. 18 ^{13}C liquid NMR spectrum of DMAP and ^{13}C solid-state MAS NMR spectrum of as-made Product B. DMAP molecules remain intact within the framework structures.

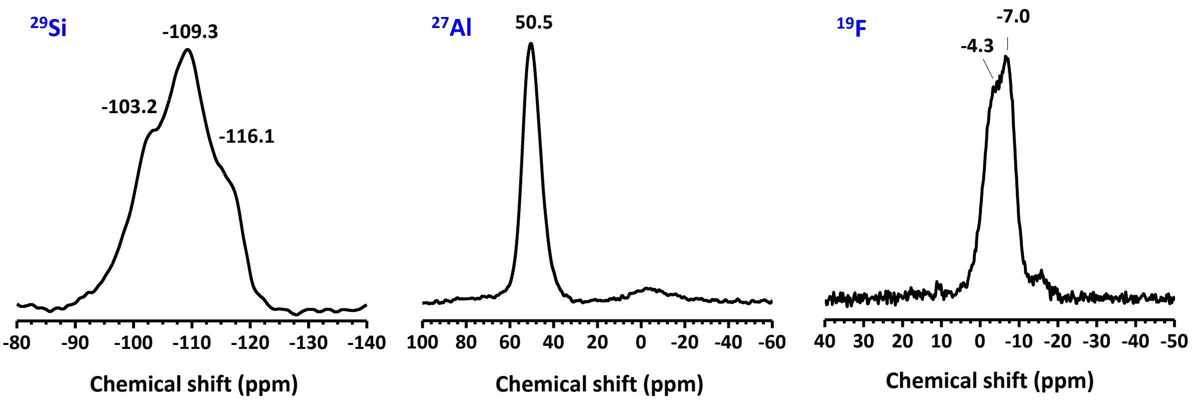

Supplementary Fig. 19 ^{29}Si, ^{27}Al, and ^{19}F solid-state NMR spectra of Product B. The spectra show that almost all Si and Al atoms are four-coordinated in the framework structures. In addition, ^{19}F NMR spectrum indicates considerable Ge atoms are located in the *d*4*r* units[2,3].

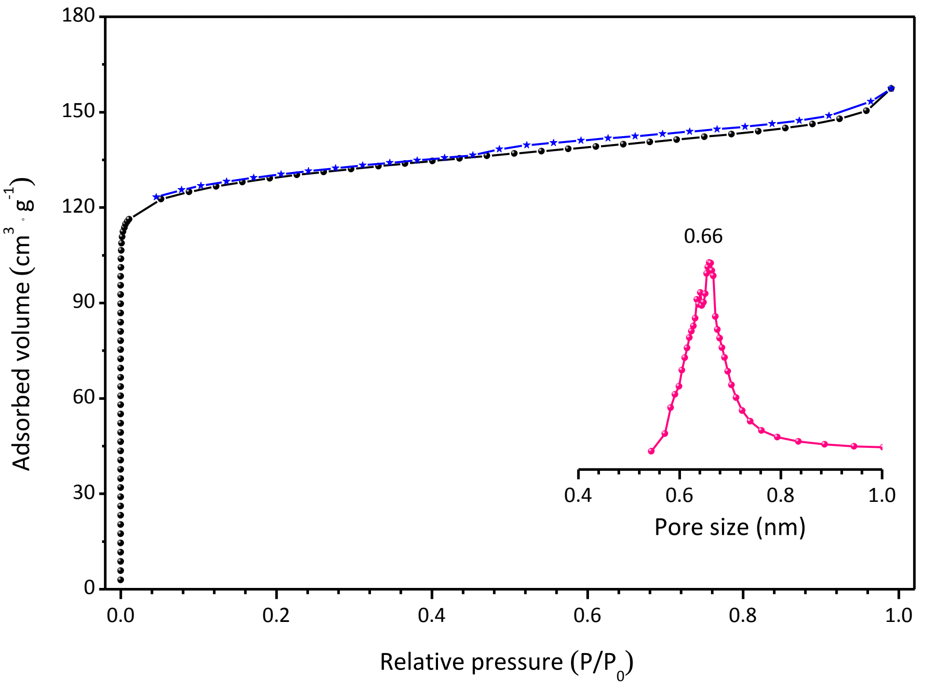

Supplementary Fig. 20 N_2 adsorption isotherms of the calcined Product B. The N_2 adsorption isotherm is shown in black spheres and the desorption isotherm is in blue stars. Inset is the pore size distribution, calculated by the Horvath-Kawazoe method[4]. Source data are available in the additional supplementary files.

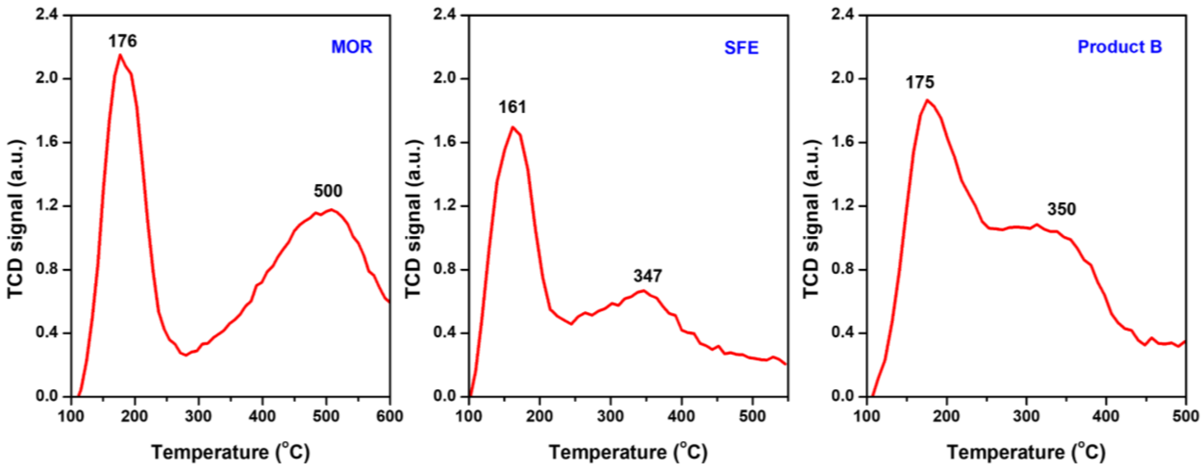

Supplementary Fig. 21 NH_3-TPD curves of MOR, SFE and Product B. The first and second TPD peaks in the curves are commonly assigned to the weak and strong acid sites of a zeolite catalyst[5]. Normally, the higher the peak temperature, the stronger the acid site. As indicated by the peak temperatures in the curves, the acid strength of **MOR** (Si/Al=18) is significantly higher than those of **SFE** (Si/B=84, Si/Al=57) and Product B (Si/Ge=6, Si/Al=17). Conversely, **SFE** and Product B possess moderate acid strength. TCD= thermal conductivity detector.

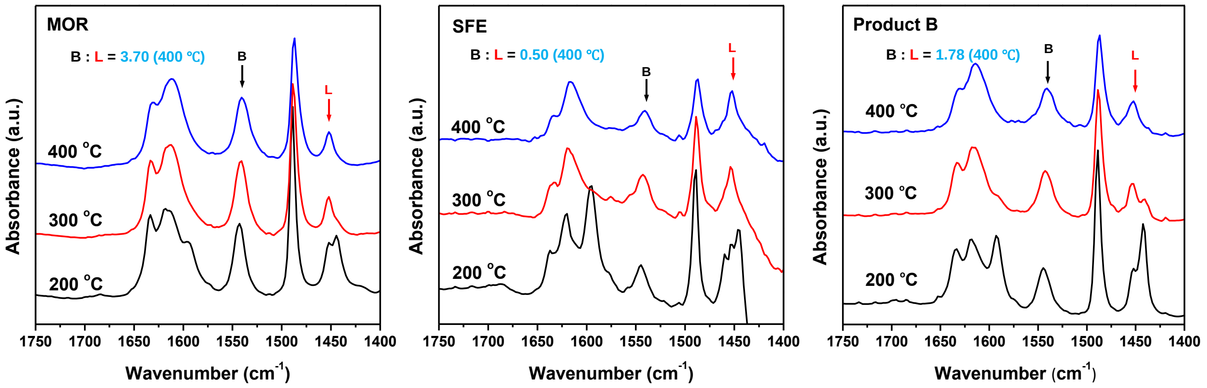

Supplementary Fig. 22 FT-IR spectra of MOR, SFE, and Product B after adsorbing pyridine followed by desorption at different temperatures. The strong acidity is dominated by Brönsted acid (B : L=3.70, 400 ℃) for **MOR** and Lewis acid (B : L=0.5) for **SFE**. While in Product B, the concentration of Brönsted acid is higher than that of Lewis acid but with a moderate ratio (B : L=1.78) compared with those of **MOR** and **SFE**.

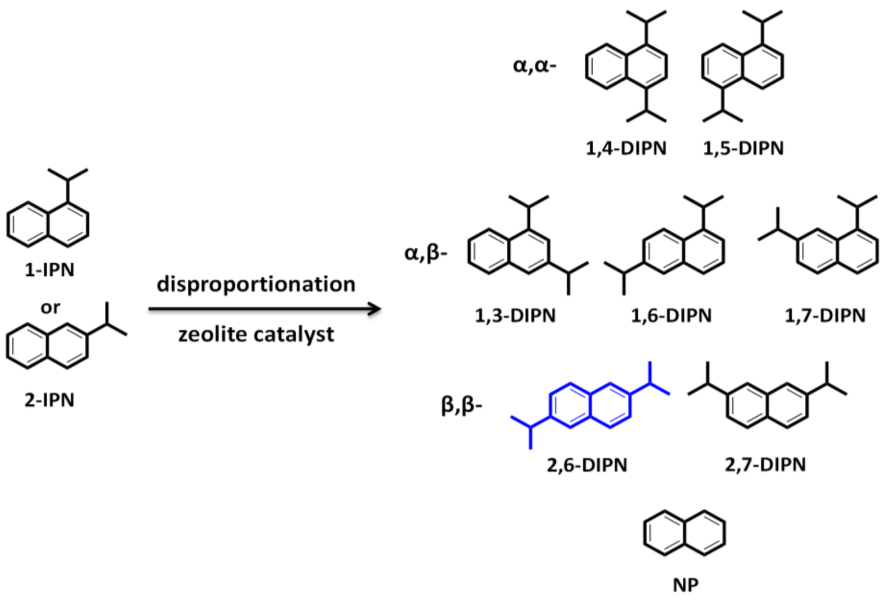

Supplementary Fig. 23 Possible products in the disproportionation of IPN. The increasing order of the critical size of DIPN molecules is 2,6-DIPN ≤ 2,7-DIPN < 1,6-DIPN ≤ 1,5-DIPN << 1,7-DIPN < 2,3-DIPN < 1,4-DIPN < 1,3-DIPN Disproportionation of the IPN is a complex reaction that produces numerous DIPN isomers, such as 1,3-DIPN, 1,7-DIPN, 2,6-DIPN (target product), and 2,7-DIPN[6,7].

Supplementary Table 1 SerialRED results of Product A from unit-cell-based clustering. The index of datasets corresponds to the index shown in Fig. 3.

Group index	Dataset index: Determined unit cell in standardized P1 setting	Rotation range	Phase
1	0 : [13.96 14.33 14.68 75.51 68.44 62.38]	20.24	**IWV**
	3 : [12.86 13.98 15.51 78.34 67.46 65.18]	59.60	
	4 : [13.13 13.91 13.96 77.10 64.10 62.92]	40.09	
	8 : [12.85 14.14 15.32 78.52 66.70 65.39]	36.15	
	9 : [12.86 13.97 14.18 79.44 67.72 64.80]	44.09	
	17 : [13.77 14.31 14.61 76.67 63.22 62.72]	66.93	
	19 : [13.95 14.01 15.88 79.01 67.37 61.07]	64.25	
	29 : [14.74 14.92 17.05 82.80 69.10 62.17]	21.85	
	32 : [13.38 14.26 14.50 77.96 66.13 63.73]	37.07	
	37 : [13.81 14.17 14.61 75.10 66.50 63.84]	35.15	
	39 : [13.88 14.41 15.74 79.93 66.14 62.00]	25.40	
	42 : [13.53 14.14 14.24 78.45 63.67 62.41]	27.41	
	46 : [12.71 13.00 14.18 84.43 67.90 64.16]	31.72	
	47 : [13.76 14.88 16.01 80.19 65.75 66.07]	59.50	
	59 : [13.70 14.29 14.79 78.61 62.57 63.15]	44.50	
	69 : [14.14 14.63 15.88 102.64 104.82 116.34][a]	25.92	
	70 : [13.20 13.82 14.13 76.28 63.05 63.31]	38.13	
	71 : [14.08 14.42 16.48 71.53 65.21 62.15]	22.46	
	72 : [14.00 13.56 16.05 78.31 66.55 61.85]	30.70	
	73 : [14.87 14.45 15.71 75.84 62.84 63.19]	31.06	
2	1 : [9.90 10.86 11.07 75.59 74.24 76.42]	22.76	**RTH**
	2 : [9.52 10.26 10.99 85.42 67.36 81.10]	22.57	
	6 : [9.81 10.11 11.53 87.83 70.59 79.88]	26.10	
	11 : [9.71 10.44 12.04 83.33 69.63 83.99]	38.84	
	16 : [9.72 10.24 10.79 101.55 90.20 90.50]	35.40	
	18 : [10.08 10.60 11.66 68.3 83.33 85.40]	40.15	
	21 : [9.24 9.46 11.11 66.93 86.98 83.08]	49.80	
	24 : [10.26 10.45 11.44 82.80 64.26 79.09]	28.27	
	25 : [9.20 9.28 10.78 83.00 65.63 83.70]	24.50	
	31 : [9.30 9.50 10.82 66.25 82.97 83.90]	26.91	
	38 : [9.44 9.87 10.95 113.64 90.51 97.71]	60.20	
	44 : [10.16 10.39 12.24 95.52 113.75 96.75]	22.06	
	49 : [9.58 10.23 12.34 88.05 70.19 84.12]	30.80	
	53 : [9.29 9.56 10.15 89.54 65.42 86.06]	31.76	
	54 : [9.67 9.67 11.13 68.90 84.10 82.80]	53.23	
	55 : [10.32 10.82 11.73 79.84 65.49 80.99]	22.59	
	58 : [9.43 9.53 11.07 92.39 112.91 94.90]	48.99	
3	5 : [4.65 12.82 14.79 64.95 88.74 87.05]	37.23	***CTH**
	13 : [4.94 13.23 14.87 64.39 85.76 88.32]	35.50	
	14 : [5.22 15.07 15.62 110.73 94.96 99.14][a]	27.21	
	23 : [4.82 14.06 15.10 64.41 87.47 89.49]	27.57	
	26 : [5.21 13.63 15.49 115.93 98.06 92.09][a]	31.25	
	27 : [5.67 13.91 15.71 64.08 89.34 87.58]	35.29	
	28 : [4.96 14.69 15.57 113.37 91.01 91.67][a]	44.09	
	30 : [5.25 14.36 15.00 66.33 87.6 84.59]	33.68	
	34 : [5.40 13.50 16.05 108.90 94.10 93.10][a]	25.00	
	35 : [5.01 13.38 14.08 64.35 85.18 86.96]	44.29	
	36 : [4.88 12.70 14.37 68.50 86.34 89.20]	49.49	
	45 : [5.52 15.08 16.23 110.81 92.17 93.60][a]	25.34	
	48 : [5.39 13.95 15.22 63.08 88.49 89.70]	26.86	
	50 : [5.13 14.71 15.72 62.13 88.50 83.10]	22.37	
	51 : [5.22 13.42 15.53 112.63 91.57 93.47][a]	32.63	
	52 : [5.17 13.51 15.56 70.00 89.41 88.98][a]	45.46	

	56 : [5.29 14.34 16.05 67.24 89.24 89.25]	35.10	
	62 : [5.46 14.46 16.05 70.34 88.89 88.68]	30.90	
	64 : [5.07 14.47 14.65 118.60 91.50 90.20]	22.42	
	67 : [5.01 13.46 14.93 64.73 86.06 86.15]	54.04	
	75 : [5.22 13.64 14.27 116.37 90.93 90.29]	22.72	
	76 : [5.28 13.98 16.47 69.38 86.59 87.62]	26.12	
4	7 : [4.92 10.75 11.03 79.79 77.78 80.18]	27.72	Unclassified
5	10 : [4.83 4.94 5.15 92.29 92.24 115.58]	22.73	SiO_2/GeO_2
	74 : [4.18 4.44 5.67 90.50 110.83 111.36]	35.35	
6	12 : [5.16 11.94 12.72 83.00 81.81 77.84]	45.30	***UOE**
	15 : [5.18 12.17 13.97 79.60 82.50 79.50]	39.96	
7	20 : [5.26 8.29 14.40 77.10 84.5 84.10]	57.84	Unclassified
	65 : [5.27 8.00 12.54 95.20 102.10 94.10]	80.17	
8	22 : [4.96 5.37 6.80 81.10 89.40 67.80]	35.60	SiO_2/GeO_2
	41 : [5.27 6.05 7.10 90.92 111.02 106.80]	31.05	
9	33 : [9.95 13.80 14.82 65.44 88.19 89.47]	26.75	Unclassified
	57 : [9.52 13.79 15.65 65.85 88.64 85.72]	23.73	Unclassified
10	40 : [10.95 18.76 19.37 89.23 87.17 87.4]	27.52	**POS**
11	43 : [4.85 15.87 15.89 117.00 92.10 93.80]	43.94	Unclassified
12	60 : [6.92 7.64 8.49 66.60 69.40 80.80]	34.96	Unclassified
13	61 : [4.75 4.83 9.90 89.81 88.29 61.97]	48.90	Unclassified
14	63 : [4.78 8.25 9.60 69.90 85.98 88.70]	37.93	Unclassified
	68 : [5.19 7.49 8.32 94.86 107.50 96.70]	63.85	
15	66 : [3.18 9.73 11.27 65.62 87.45 87.35]	49.55	Unclassified

[a] unit cell with supplementary angles

In order to make unit cell dimensions from datasets with different data quality comparable, the reduced unit cell from *DIALS* in *P*1 setting is always extracted[8]. This is to eliminate the unit cell difference that comes merely from the transformation with lattice symmetry. The distance metric used in the clustering is defined in Eq. 1 in the Methods section.

The deviations of unit cell parameters can be quite big. This can be mainly ascribed to the hight change of the crystal, shift of the direct beam, and relatively low rotation range. The unclassified unit cells mainly associate to datasets of multiple lattices or with poor data quality, resulting in unit cell parameters with large errors or being wrong.

Supplementary Table 2 Primitive unit cells and the corresponding unit cells with the highest symmetry of different zeolites. It is worth mentioning that the crystals of ***CTH** and ***UOE** contain stacking disorders along the *a*-axis. The *a*-parameter here represents an average value where diffuse scattering has been ignored, resulting in half of the *a*-parameter in the ordered structure model.

Phase	Primitive unit cell parameters						Space group
**RTH**	*a*=10.3Å	*b*=10.5 Å	*c*=11.6 Å	α=89.0°	*β*=65.7°	γ=85.0°	*P1*
**IWV**	*a*=14.4 Å	*b*=14.5 Å	*c*=15.5 Å	α=77.8°	*β*=63.0°	γ=62.0°	*P1*
***CTH**	*a*=5.0 Å	*b*=13.8 Å	*c*=15.0 Å	α=65.0°	*β*=90.0°	γ=90.0°	*P1*
**POS**	*a*=11.9 Å	*b*=19.6 Å	*c*=19.6 Å	α=90.0°	*β*=90.0°	γ=90.0°	*P1*
***UOE**	*a*= 5.3 Å	*b*=11.8 Å	*c*=12.0 Å	α=80.0°	*β*=79.0°	γ=79.0°	*P1*
Phase	Unit cells with the highest symmetry						Space group
**RTH**	*a*=10.3 Å	*b*=21.2 Å	*c*=10.5 Å	α=90.0°	*β*=95.0°	γ=90.0°	*C2/m*
**IWV**	*a*=14.4 Å	*b*=25.6 Å	*c*=27.6 Å	α=90.0°	*β*=90.0°	γ=90.0°	*Fmmm*
***CTH**	*a*=5.0 Å	*b*=27.2 Å	*c*=13.8 Å	α=90.0°	*β*=90.0°	γ=90.0°	*Cmmm*
**POS**	*a*=19.6 Å	*b*=19.6 Å	*c*=11.9 Å	α=90.0°	*β*=90.0°	γ=90.0°	$P4_2/mnm$
***UOE**	*a*=5.3 Å	*b*=15.3 Å	*c*=17.7 Å	α=90.0°	*β*=90.0°	γ=90.0°	*Immm*

Supplementary Table 3 Crystallographic information of the three major phases in Product A that were solved using SerialRED data. The crystal of ***CTH** contains stacking disorder and only the average structure is obtained.

Phase	**RTH**	**IWV**	***CTH**
Unit cell (Å)	9.68, 20.83, 10.00, β=95.4°	27.83, 26.08, 13.94	13.69, 27.50, 5.04
Space group	$C2/m$	$Fmmm$	$Cmm2$
Resolution (Å)	1.0	0.8	0.8
$CC_{1/2}$ (%) overall	98.8	96.7	97.7
$CC_{1/2}$ (%) (highest resolution shell)	65.7 (1.1-1.0 Å)[a]	79.8 (0.82-0.80 Å)[a]	61.2 (0.84-0.80 Å)[a]
I/σ overall	2.78	2.68	4.46
I/σ (highest resolution shell)	1.72 (1.1-1.0 Å)[a]	1.19 (0.82-0.80 Å)[a]	2.39 (0.84-0.80 Å)[a]
Completeness (%)	52.0 (5 datasets)	97.6 (8 datasets)	79.9 (5 datasets)
$Rint/R1/wR2$ [F^2 >2.0σ(F^2)]	0.1238/0.2736/0.6111	0.2908/0.3165/0.6678	0.1375/0.2165/0.5162
No. of reflections (observed)	624 (397)	2549 (1202)	1565 (1135)
No. of parameters (restraints)	113 (108)	153 (218)	69 (25)

[a] The high $CC_{1/2}$ and I/σ values are because a high threshold was used for the resolution cut and the data merging. Due to the scaling in data merging, the $CC_{1/2}$ and I/σ values of the merged data can be higher than those of individual datasets.

Supplementary Table 4 SerialRED results of Product B from unit-cell-based clustering. The index of datasets corresponds to the index shown in Supplementary Fig. 13.

Group index	Dataset index: Determined unit cell in standardized P1 setting	Rotation range	Phase
1	0 : [13.51 13.96 15.53 66.85 82.20 68.18]	37.80	**IWV**
	1 : [13.51 14.41 14.79 81.25 66.51 66.49]	22.93	
	2 : [13.99 13.99 14.89 76.67 68.88 62.50]	43.76	
	3 : [13.76 14.99 15.56 80.70 63.03 66.01]	39.51	
	4 : [15.03 15.50 16.50 69.78 61.75 78.47]	21.47	
	7 : [13.95 14.55 16.38 80.73 70.94 62.66]	29.27	
	9 : [13.77 13.79 16.45 81.07 64.99 63.98]	35.97	
	14 : [14.19 14.30 16.58 80.73 68.22 62.64]	29.31	
	17 : [13.95 14.67 15.01 78.80 65.03 66.41]	34.20	
	18 : [14.25 14.63 16.28 67.77 77.93 66.24]	21.09	
	19 : [13.94 14.45 15.16 81.28 70.52 62.11]	51.98	
	20 : [13.65 14.76 15.78 61.56 78.18 65.66]	39.41	
	21 : [14.31 14.39 16.17 76.51 65.97 65.74]	30.38	
	23 : [15.01 15.17 15.53 79.83 77.61 63.55]	33.90	
	28 : [13.79 14.90 17.60 81.22 67.33 63.35]	34.16	
	31 : [13.91 14.44 14.96 73.89 67.53 62.84]	30.77	
	40 : [15.27 15.38 16.68 81.94 71.91 60.21]	41.48	
	42 : [14.56 14.66 16.95 76.01 66.74 60.97]	47.65	
	43 : [13.57 14.08 14.41 81.33 68.94 61.98]	39.61	
	45 : [14.02 14.05 14.85 62.52 74.19 62.44]	50.67	
	49 : [14.32 14.62 16.12 78.48 68.78 66.26]	21.32	
	51 : [14.63 14.73 14.82 63.91 74.74 60.53]	31.07	
	56 : [14.10 15.44 16.43 79.29 68.67 76.94]	44.21	
	57 : [14.58 14.93 15.03 80.57 61.18 69.27]	58.50	
	61 : [13.78 13.84 15.41 105.25 100.76 117.26]	38.14	
	62 : [14.07 14.48 16.43 68.05 77.96 62.02]	34.06	
	64 : [13.09 13.73 14.87 84.39 65.30 66.94]	36.94	
2	5 : [11.09 13.21 14.34 93.21 93.86 110.60]	26.89	Unclassified
	53 : [10.79 12.68 12.89 48.11 86.65 87.99]	51.78	
3	6 : [10.74 15.29 19.96 105.53 85.02 107.55]	42.33	Unclassified
	25 : [9.94 13.53 20.78 50.55 79.24 82.82]	35.31	
4	8 : [5.08 15.23 17.79 70.44 84.59 89.18]	41.83	***CTH**
	10 : [4.95 13.80 16.77 101.83 86.54 88.43]	26.28	
	11 : [5.27 14.15 15.28 70.55 90.90 87.64]	34.92	
	12 : [5.14 14.58 15.67 114.09 89.47 95.63]	25.53	
	16 : [4.97 14.04 14.91 69.14 85.24 88.08]	43.55	
	35 : [5.02 14.05 15.68 112.54 93.18 91.99]	30.16	
	37 : [5.57 14.97 16.74 114.79 102.06 84.89]	22.44	
	46 : [5.30 15.35 17.07 105.90 93.19 94.77]	26.47	
	50 : [5.29 13.52 15.33 66.19 87.58 81.43]	60.33	
5	13 : [20.14 20.36 24.79 96.29 73.85 70.27]	47.80	Unclassified
	39 : [18.79 21.92 25.65 88.53 97.53 114.22]	58.45	
6	15 : [0.01 12.22 15.30 66.74 89.94 89.85]	30.47	Unclassified
7	22 : [14.46 16.59 18.80 59.27 68.99 79.43]	44.20	Unclassified
	44 : [14.20 17.88 18.42 111.72 107.70 92.18]	40.37	
8	24 : [14.63 15.24 20.86 94.56 86.30 56.49]	20.36	Unclassified
	27 : [14.13 14.43 19.86 82.43 69.88 62.55]	38.45	
	32 : [13.52 15.50 21.42 100.51 104.71 114.75]	68.51	
	33 : [13.98 14.52 19.29 83.77 69.05 63.05]	33.70	
	58 : [13.91 14.52 19.96 82.31 61.71 63.71]	23.64	
9	26 : [8.35 11.33 11.66 71.60 73.41 83.14]	43.71	Unclassified
	41 : [8.67 11.35 13.37 83.74 76.60 84.45]	39.46	

10	29 : [7.41 12.04 64.22 147.04 75.44 113.72]	22.77	Unclassified
11	30 : [5.12 5.79 5.86 87.51 89.23 65.10]	34.97	Unclassified
	47 : [3.85 4.19 4.22 60.64 85.22 61.93]	23.09	
	54 : [5.20 5.21 5.56 87.08 87.11 66.27]	30.52	
12	34 : [5.22 25.00 26.77 115.78 96.07 92.99]	35.41	Unclassified
13	36 : [14.21 15.79 28.12 67.22 76.55 64.11]	28.49	Unclassified
	59 : [14.59 14.65 27.21 94.49 83.33 63.57]	31.12	
14	38 : [5.42 7.72 15.38 80.17 80.53 70.60]	31.28	Unclassified
	48 : [5.95 7.32 13.30 92.78 95.31 112.25]	25.43	
15	52 : [14.21 14.95 39.12 76.89 88.34 64.27]	20.87	Unclassified
16	55 : [14.11 15.11 55.55 84.82 84.49 62.29]	33.81	Unclassified
17	60 : [14.22 14.91 24.28 80.09 69.04 62.20]	35.83	Unclassified
18	63 : [15.26 18.61 26.54 80.57 93.69 81.96]	47.91	Unclassified

The unclassified unit cells mainly associate to datasets of multiple lattices or poor data quality, resulting in unit cell parameters with large errors or being wrong.

Supplementary Table 5 Crystallographic information of the major phase IWV in Product B determined using the SerialRED data. The data completeness of the phase ***CTH** is too low (56.2% with a resolution of 0.8 Å) for the structure solution.

Phase	**IWV**
Unit cell (Å)	28.82, 25.75, 13.90
Space group	*Fmmm*
Resolution (Å)	0.9
$CC_{1/2}$ (%) overall	90.2
$CC_{1/2}$ (%) (highest resolution shell)	91.8(0.92-0.90 Å)[a]
I/σ overall	5.14
I/σ (highest resolution shell)	2.90 (0.92-0.90 Å)
Completeness (%)	99.7 (11 datasets)
$Rint/R1/wR2$ [F^2 >2.0σ(F^2)]	0.2647/0.2076/0.4767
No. of reflections (observed)	1875 (1491)
No. of parameters (restraints)	151 (32)

[a]The $CC_{1/2}$ value in the outer resolution shell is slightly higher than the overall $CC_{1/2}$. This is because the data was cut at a resolution (0.9 Å) where the I/σ is still very high (2.9) and the $CC_{1/2}$ value remains also high. Considering the noise, the $CC_{1/2}$ value in the outer resolution shell happens to be slightly higher than the overall $CC_{1/2}$ value.

Supplementary Table 6 Crystallographic data and parameters of Rietveld refinement against the SPXRD data of the phase in the calcined Product B.

Parameters	**IWV**	***CTH**
Phase composition (%)	67	33
Chemical Formula[a]	$[Si_{136}Ge_{16}O_{304}]$	$[Si_{28}Ge_4O_{64}]$
Space group	$Fmmm$	$Cmm2$
a (Å)	13.7812(4)	13.7200(5)
b (Å)	25.2863(10)	27.5719(13)
c (Å)	28.1442(10)	5.0376(3)
V (Å^3)	9807.6(6)	1905.65(15)
2θ range (°)	1.8 to 35.0	
Wavelength (Å)	0.68950 (synchrotron)	
R_I	0.027 (**IWV**), 0.033 (***CTH**)	
R_{wp}	0.169	
R_{exp}	0.116	
GoF	1.45	
Observations	9804	
Contributing reflections	1214 (**IWV**), 529 (***CTH**)	
Total parameters	152	
Restraints	84 (**IWV**), 74 (***CTH**)	

[a] The main purpose of the Rietveld refinement conducted here is to determine the phase composition of Product B. Al atoms were not included during the refinement as it is challenging to distinguish Si and Al using the SPXRD data. Ge atoms were included in refinement because of the larger difference in the atomic scattering factors of Ge and Si. The preferred locations of Ge atoms could be identified, which are in the *d*4*r* units. The ^{19}F solid-state NMR spectrum also shows that significant Ge atoms are located in the *d*4*r* units (Supplementary Fig. 19).

Supplementary Table 7 The reported expensive, bulky, and complex OSDAs that were used to synthesize SFE, IWV, and *CTH.

Zeolite	OSDA	Reference
**SFE**	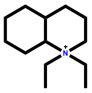 *N*,*N*-Diethyldecahydroquinolinium	1,9
**IWV**	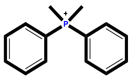 dimethyldiphenylphosphonium 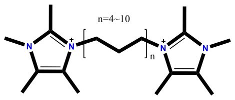 diquaternary imidazolium	10,11
***CTH**	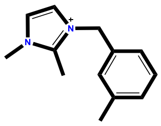 1,2-Dimethyl-3-(3,5-dimethylbenzyl)imidazolium 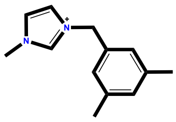 1-Methyl-3-(3,5-dimethylbenzyl)imidazolium	12

Supplementary Table 8 Textural properties of MOR, SFE, and Product B

Sample	Si/Ge[a]	Si/B[a]	Si/Al[a]	S_{BET}[b] (m^2/g)	S_{micro}[b] (m^2/g)	S_{ext}[b] (m^2/g)	V_{micro}[b] (cm^3/g)
**MOR**	∞	∞	18	373	348	25	0.16
**SFE**[c]	∞	84	57	375	241	134	0.11
Product B	6	∞	17	427	368	59	0.17

[a] Analyzed by ICP. [b] Analyzed by BET and t-plot methods.

[c] SFE zeolite was first synthesized in borogermanosilicate form using DMAP as the OSDA. Its aluminoborosilicate form was prepared later by optimizing the synthesis condition[13].

Supplementary Table 9 Catalytic results of IPN disproportionation over MOR, SFE and Product B catalysts. The reaction time was 6 hours and the temperature was 250 ℃.

Sample	IPN conv. (%)[a]	Distribution of diisopropylnaphthalenes (DIPN) (mol %)					β,β-selectivity (mol %)[b]	2,6-DIPN/ 2,7-DIPN
		1,3-DIPN	1,7-DIPN	2,6-DIPN	2,7-DIPN	Other		
**MOR**	15.3	11.1	5.2	45.5	29.8	8.3	75.3	1.5
**SFE**	6.4	21.8	13.5	37.9	17.3	9.5	55.2	2.2
Product B	32.3	4.8	6.6	42.2	37.6	8.8	79.8	1.1

[a] IPN=isopropylnaphthalene. [b] *β,β*-selectivity=(2,6-DIPN+2,7-DIPN)/DIPN*100%.

Supplementary Table 10 The ideal bond lengths and angles of different framework T atoms[14-16]

T_1-O-T_2	r (T_1-O)/Å	r (T_2-O)/Å	∠T_1-O-T_2/°
Si-O-Si	1.61	1.61	145
Si-O-Al	1.61	1.70	138
Si-O-B	1.61	1.35	129
Si-O-Ge	1.61	1.74	138
Ge-O-Ge	1.74	1.74	133

Supplementary notes

1.1 Instructions for *Instamatic*

Instamatic is a Python package that is being developed with the aim to automate the collection of electron diffraction data. At the core is a Python library for transmission electron microscope experimental control with bindings for the JEOL/FEI microscopes and interfaces to the ASI/TVIPS/Gatan cameras. Routines have been implemented for collecting continuous rotation electron diffraction (cRED, 3D ED, or microED), serial electron diffraction (SerialED), serial rotation electron diffraction (SerialRED) data, and stepwise rotation electron diffraction (RED) data. The source code of *Instamatic* package is available at https://github.com/instamatic-dev/ilnstamatic. The hardware and software requirements for installation and running of *Instamatic* are described in the *readme.md* file (https://github.com/instamatic-dev/instamatic/blob/master/readme.md). Documentation of *Instamatic* package is available at https://instamatic.readthedocs.io, where more details of the program, setting up *Instamatic*, examples of running *Instamatic* for data collection, API reference, etc., are presented. For the details of setting up SerialRED experiment, users can refer to our previous paper (Automated serial rotation electron diffraction combined with cluster analysis: an efficient multi-crystal workflow for structure determination. *IUCrJ* **6**, 854–867 (2019)).

1.2 Instructions for *edtools*

edtools is a python package of a collection of tools for automated processing of batch 3D ED datasets. The source code of *edtools* (version: 1.0.4) is available at https://doi.org/10.5281/zenodo.6952810. The requirements for installation and running *edtools* are given in the *readme.md* file (https://github.com/instamatic-dev/edtools/blob/master/README.md). Documentation of *edtools* package is available at https://edtools.readthedocs.io, where one can find more details of the program, installation of *edtools*, a demo of running *edtools* for automated processing of batch 3D ED datasets, API reference, etc. Users can find more details regarding *edtools* in our previous paper. (Automated serial rotation electron diffraction combined with cluster analysis: an efficient multi-crystal workflow for structure determination. *IUCrJ* **6**, 854–867 (2019)).

For running *edtools*, the *XDS* package for reduction of 3D ED datasets is required. *XDS* package is available at https://xds.mr.mpg.de/html_doc/downloading.html. The instruction for the installation of *XDS* package can be found at https://strucbio.biologie.uni-konstanz.de/xdswiki/index.php/Installation. A typical cycle of using *edtools* for processing batch 3D ED datasets for phase analysis and structure determination goes through the following steps: 1) edtools.autoindex, 2) edtools.extract_xds_info, 3) edtools.find_cell, 4) edtools.update_xds, 5) edtools.make.xscale, and 6) edtools.cluster. A step-by-step demonstration of processing batch 3D ED datasets is as following:

1.2.1 Automated indexing

Before running *edtools*, make sure that you have put all your 3D ED datasets in one folder. All the 3D ED datasets are expected to be in the same *XDS* readable image format, e.g. `SMV`. A correctly

configured XDS.INP file is also expected for each dataset. Then open the windows command prompt from the root directory which contains all the datasets. Automatic indexing for batch 3D ED datasets is realized by running *XDS* in all subfolders (SMV) that contain file XDS.INP, which is automatically generated during data collection by *Instamatic*.

Command: edtools.autoindex
-In: XDS.inp
-Out: XDS data processing on all files

1.2.2. Extract unit cell information

Extract the determined unit cell parameters from the output files (CORRECT.LP) of *XDS*.

Command: edtools.extract_xds_info
-In: CORRECT.LP
-Out: cells.yaml
      cells.xlsx
      filelist.txt

1.2.3 Unit-cell-based clustering for phase analysis

The primitive unit cells in the cells.yaml file are clustered with the metric lcv. The distance cut-off can be selected by clicking in the interactive dendrogram plot. The unit cells belonging to each phase will be written to the corresponding cells_cluster_*.yaml file.

Command: edtools.find_cell cells.yaml -s --cluster --metric lcv
-In: cells.yaml
-Out: mean unit cell parameters of each cluster
      cells_*-items.yaml

1.2.3 Update the XDS.INP files

With the averaged primitive unit cell parameters of each phase, one can use the online tool http://cci.lbl.gov/cctbx/lattice_symmetry.html to find unit cell with higher symmetry with a preset tolerance. This step is to update the XDS.INP files with the determined unit cell parameters and space group. The same operation can be done for all the phases.

Command: edtools.update_xds -c _ _ _ _ _ _ -s _ (replace the underscore with the unit cell parameters and space group)
-In: unit cell parameters and space group / XDS.INP
-Out: updated XDS.INP

1.2.4 Refine phases

Rerun commands edtools.autoindex, edtools.extract_xds_info, and edtools.find_cell for the desired phases to be successfully indexed by *XDS*. All the other phases are hopefully excluded in that a phase with a different enough unit cell will not be indexed successfully. There are however cases when different phases have similar unit cells, which cannot be told apart during this step.

Command: `edtools.autoindex`
-In: XDS.inp
-Out: XDS data processing on all files
Command: `edtools.extract_xds_info`
-In: CORRECT.LP
-Out: cells.yaml
      cells.xlsx
      filelist.txt
Command: `edtools.find_cell cells.yaml -s --cluster --metric lcv`
-In: cells.yaml
-Out: mean unit cell parameters of each cluster
      cells_*-items.yaml

1.2.5 Generate the input file for XSCALE

This command generates the desired unit cell cluster for *XSCALE*.

Command: `edtools.make_xscale cells_cluster_*-items.yaml -c _ _ _ _ _ _ -s _` (replace the underscore with the unit cell parameters and space group)
-In: cells_cluster_*-items.yaml / XDS_ASCII.HKL
-Out: XSCALE.INP
      XDSCONV.INP

1.2.6 Run *XSCALE*

XSCALE calculates the correlation coefficients between different datasets.

Command: `wsl xscale`
-In: XSCALE.INP
-Out: XSCALE.LP

1.2.7 Intensity-based clustering

Run intensity-based clustering to further filter out datasets with low correlation (to remove poor quality datasets), or from a different phase with a similar enough unit cell. Cut-off on the dendrogram is selected manually. A number below 0.4 can be a good starting choice. In the end, integration results from datasets corresponding to different clusters are automatically copied to different folders after running clustering. The merged intensities in file `shelx.hkl` can be used for structure determination.

Command: `edtools.cluster`
-In: XSCALE.LP
-Out: cluster_n/
            filelist.txt
            *_XDS_ASCII.HKL
            XSCALE processing
            pointless processing
            shelx.hkl

The step-by-step demo with more information is available at https://edtools.readthedocs.io/en/latest/examples/edtools_demo.html.

References

1. Lee, G. S. *et al.* Organocations in zeolite synthesis: fused bicyclo [l.m.0] cations and the discovery of zeolite SSZ-48. *J. Am. Chem. Soc.* **124**, 7024–7034 (2002).
2. Corma, A. *et al.* Extra-large pore zeolite (ITQ-40) with the lowest framework density containing double four- and double three-rings. *PNAS* **107**, 13997–14002 (2010).
3. Verheyen, E. *et al.* Design of zeolite by inverse sigma transformation. *Nat. Mater.* **11**, 1059–1064 (2012).
4. Horváth, G. & Kawazoe, K. Method for the calculation of effective pore size distribution in molecular sieve carbon. *J. Chem. Eng. Jpn* **16**, 470–475 (1983).
5. Lónyi, F. & Valyon, J. On the interpretation of the NH_3-TPD patterns of H-ZSM-5 and H-mordenite. *Micropor. Mesopor. Mater.* **47**, 293–301 (2001).
6. Brzozowski, R. Isomerization of diisopropylnaphthalenes on wide-pore zeolites. *J. Catal.* **232**, 366–377 (2005).
7. Schmitz, A. D. & Song, C. Shape-selective isopropylation of naphthalene. Reactivity of 2,6-diisopropylnaphthalene on dealuminated mordenites. *Catal. Today* **31**, 19–25 (1996).
8. Winter, G. *et al.* DIALS: implementation and evaluation of a new integration package. *Acta Cryst.* **D74**, 85–97 (2018).
9. Wagner, P. *et al.* Electron diffraction structure solution of a nanocrystalline zeolite at atomic resolution. *J. Phys. Chem. B* **103**, 8245–8250 (1999).
10. Dorset, D. L. *et al.* P-derived organic cations as structure-directing agents: synthesis of a high-silica zeolite (ITQ-27) with a two-dimensional 12-ring channel system. *J. Am. Chem. Soc.* **128**, 8862–8867 (2006).
11. Schmidt, J. E., Chen, C.-Y., Brand, S. K., Zones, S. I. & Davis, M. E. Facile synthesis, characterization, and catalytic dehavior of a large-pore zeolite with the IWV framework. *Chem. Eur. J.* **22**, 4022–4029 (2016).
12. Kang, J. H. *et al.* Synthesis and characterization of CIT-13, a germanosilicate molecular sieve with extra-large pore openings. *Chem. Mater.* **28**, 6250–6259 (2016).
13. Luo, Y. *et al.* A facile and green method for the synthesis of SFE borosilicate zeolite and its heteroatom-substituted analogues with promising catalytic performances. *Chem. Eur. J.* **24**, 306–311 (2018).
14. Zones, S. I. Translating new materials discoveries in zeolite research to commercial manufacture. *Micropor. Mesopor. Mater.* **144**, 1–8 (2011).
15. Wragg, D. S., Morris, R. E. & Burton, A. W. Pure silica zeolite-type frameworks: a structural analysis. *Chem. Mater.* **20**, 1561–1570 (2008).

16. Sastre, G. & Corma, A. Rings and strain in pure silica zeolites. *J. Phys. Chem. B* **110**, 17949–17959 (2006).
